# Tactile perception of roughness, texture, and friction on compliant surfaces

**DOI:** 10.3758/s13414-026-03361-1

**Published:** 2026-11-11

**Authors:** Maja Fehlberg, Roland Bennewitz

**Affiliations:** 1 INM - Leibniz Institute for New Materials, Campus D2 2, 66123 Saarbrücken, Germany; 2 Department of Physics, Saarland University, Campus, 66123 Saarbrücken, Germany

**Keywords:** Roughness perception, Surface texture, Friction perception, Duplex theory, Fingertip

## Abstract

Skin friction and surface texture are critical tactile cues through which the somatosensory system differentiates and interprets materials. We present three psychophysical experiments on roughness sorting, texture rating, and friction discrimination with randomly rough and microstructured polyurethane rubber samples. Participants agreed to a high degree in their roughness sorting within and between both surface-texture types. We identified two sample clusters in the microstructured sample set with different texture perception under static and dynamic exploration. Additionally, we found a higher just-noticeable difference for friction discrimination between samples from different sample clusters, compared to samples from the same cluster. The separation into two sample clusters with microstructure sizes smaller and larger than 100 µm suggests that the duplex theory of texture perception extends to compliant textures and that both roughness and friction perception are influenced by spatial and temporal cues. The data for all three experiments are available at https://doi.org/10.5281/zenodo.20271426. None of the experiments were preregistered.

**Supplementary Information:**

The online version contains supplementary material available at https://doi.org/10.3758/s13414-026-03361-1.

## Introduction

Touch plays a crucial role in how we perceive material properties during everyday interactions. When we run our fingers across the surface of a fabric, explore the texture of a wooden table, or hold a smartphone, we rely on tactile information to identify, differentiate, and evaluate materials.

Researchers have applied multidimensional scaling analyses to the results of psychophysical studies to explore how people describe the tactile qualities of surfaces. Initially, three dimensions were found, which were named rough/smooth, soft/hard, and sticky/slippery (Hollins, Bensmaia, Karlof, et al., 2000). A meta-analysis of tactile perception studies revealed two additional dimensions, so that we now consider five dimensions: fine and macro roughness, hardness, warmness, and friction (Okamoto, Nagano, & Yamada, 2013). Together, these dimensions capture important aspects of how humans perceive materials through touch. Here, we focus on roughness and friction and their role in the perception of surfaces with stochastic roughness and with regular micro-scale texture.

### Roughness perception

Roughness is an important tactile dimension that influences the discriminative perception of materials, for example by dominating the perception of similarity between surfaces (Bensmaia & Hollins, 2005; Bergmann Tiest & Kappers, 2007; Hollins et al., 2000; Sahli, Prot, Wang, et al., 2020).

The quantification of surface roughness varies across scientific fields; as a result, interdisciplinary haptic research lacks a unified framework for describing the physical roughness of materials. Some investigations rely on standardized profile-based parameters such as $R_a$ or $R_q$, which describe stochastic height variations with irregular or statistical distributions typically found on natural surfaces or surfaces with standard technical finish. Several studies have shown that perceived roughness correlates well with these conventional metrics (Bergmann Tiest & Kappers, 2007; Sahli et al., 2020; Skedung, Danerlöv, Olofsson, et al., 2011).

Other lines of haptic research focus on fabricated surfaces with well-defined, often periodic microstructures (Blake, Hsiao, & Johnson, 1997; Klatzky & Lederman, 1999; Lederman & Taylor, 1972; Tymms, Zorin, & Gardner, 2018). These textures are not characterized by random height statistics but by asperity shapes and sizes or pattern geometries that play a critical role in roughness perception. Increasing the spacing between surface elements tends to increase the perceived roughness up to a certain limit, beyond which the perceptual effect saturates or even declines (Blake et al., 1997; Klatzky & Lederman, 1999; Smith, Chapman, Deslandes, et al., 2002; Tymms, Zorin, & Gardner, 2018). Irregular distributions of asperities are judged to be rougher than periodic arrangements of identical asperity sizes, underscoring the importance of surface patterns for tactile perception (Tymms et al., 2018).

These studies have primarily focused on rigid surfaces, where asperity deformation under touch is negligible. In contrast, the perception of roughness in compliant materials, whose surface topography may change upon contact due to the bending or deformation of asperities, has received little attention. Textile materials are one example of such compliant surfaces. Indentation and sliding experiments on hairy textile surfaces have shown that surface elements bend, reorient, and entangle during contact, leading to contact mechanics that fundamentally differ from those of rigid rough surfaces (Camillieri & Bueno, 2015; Kyriazis, Caykara, Hefft, et al., 2026). In line with this, perceived similarity between arrays of bendable microfibrils has been reported to scale with the bending stiffness of the fibrils (Gedsun, Sahli, Meng, et al., 2022). As a result, tactile exploration of compliant materials involves a complex interaction between the skin and the surface texture, which is difficult to understand with conventional contact mechanics models.

In material science, roughness is quantified through physically measured parameters such as the root-mean-square (RMS) roughness $R_q$, the mean peak-to-valley height $R_z$, the mean surface slope $R_{\Delta q}$, and power spectral densities (PSDs), which capture specific geometric features of the surface profile. In psychophysics and perceptual research, terminology is shaped by human perception rather than standardized metrics. This study aims to contribute to clarification by studying and comparing both surfaces with random roughness and regular microstructure. We will define our use of the terms fine and coarse roughness in the light of the duplex theory of texture perception.

### Duplex theory of tactile texture perception

David Katz introduced an important hypothesis in the field of tactile texture perception, contributing significantly to its development. In his book *The world of touch*, he emphasized the role of movement in perception, as certain tactile aspects of a surface become available only in active exploration (Katz and Krueger, 2016). He introduced vibration as a temporal cue that emerges only during movement, and suggested that spatial and temporal cues have different importance for different sizes of features in the texture. This hypothesis is the conceptual foundation for what was later confirmed as the duplex theory of texture perception. In psychophysical and neurophysiological studies, a critical transition around particle sizes of 100 µm was found, below which vibrotactile cues dominate texture perception and above which spatial stimuli become the primary source of texture information (Bensmaia and Hollins, 2003; Hollins and Risner, 2000).

For coarse textures, roughness perception is mediated by spatial cues which are primarily encoded by the impulse rate of slowly adapting Merkel cells (SA-I), which respond to skin deformation (Blake et al., 1997; Connor and Johnson, 1992; Lederman, 1974; Lederman and Taylor, 1972). Roughness of coarse structures is perceived similarly well during static and dynamic exploration (Hollins and Risner, 2000), and increased normal force enhances roughness perception (Lederman and Taylor, 1972). Variations in exploration speed influence the vibration frequency but neither the speed (Lederman, 1974) nor the vibration frequency (Lederman, Loomis, & Williams, 1982) affect the perception of coarse roughness. It thus appears that temporal cues do not contribute substantially to the perception of coarse textures.

For fine textures, roughness perception relies mainly on vibrotactile information, encoded by fast-adapting Pacinian corpuscles (FA-II) which are sensitive to high-frequency vibrations (Hollins, Bensmaia, & Washburn, 2001; Vallbo, Olsson, Westberg, & Clark, 1984; Westling & Johansson, 1987). While coarse textures can be perceived with static touch, dynamic touch is required for the perception of fine textures (Hollins and Risner, 2000). Tangential movement across the surface elicits vibrations in the skin that depend on sliding speed and the spatial period of the texture (Bensmaia & Hollins, 2003; Boundy-Singer, Saal, & Bensmaia, 2017). Although exploration speed affects the vibration frequency, it was found that the vibration intensity and not the frequency reliably predict the perception of fine roughness (Bensmaia and Hollins, 2003). We test whether the duplex theory applies to compliant microstructured surfaces by using a sample set that includes structures smaller and larger than the threshold of 100 µm. We therefore intentionally selected textures from different size ranges to represent the two texture regimes proposed by the duplex theory. To test whether the duplex theory also extends to the perception of friction, we used a friction discrimination task rather than asking participants directly about texture.

### Friction perception

Friction, in both static grip and dynamic sliding, is a key tactile dimension that enables precise object manipulation, discrimination, and affection in touch. While grip force stabilizes the contact and prevents slipping, dynamic friction provides critical sensory information for surface discrimination (AliAbbasi, Aydingul, Sezgin, et al., 2023; Arvidsson, Ringstad, Skedung, et al., 2017; Gueorguiev, Bochereau, Mouraux, et al., 2016) and also contributes to the perceived pleasantness of touch (Klöcker, Wiertlewski, Théate, et al., 2013; Ramalho, Szekeres, & Fernandes, 2013; Saikumar, Fehlberg, Goktepe, et al., 2025).

Fingertip friction is determined not only by material properties, but also depends on skin characteristics and on the exploratory movements. Higher levels of skin hydration and greater skin deformability are both associated with increased friction during tactile interaction (Infante, Fehlberg, Saikumar, et al., 2025; Liu, Gad, Lu, et al., 2015; Sergachev, Matthews, & van der Heide, 2019). Parameters of tactile exploration with relevance for friction include normal force (Inoue, Okamoto, Akiyama, et al., 2022), sliding velocity (Fehlberg, Monfort, Saikumar, et al., 2024) or sliding direction (Zhang, Mo, Xu, et al., 2017).

Frictional interactions during touch deform the skin and activate cutaneous mechanoreceptors. Insights into the neural encoding of these mechanical events largely come from microneurography studies. These studies show that the onset of partial slip between skin and surface is detected by all types of tactile afferents, though fast-adapting Meissner corpuscles (FA-I) respond most prominently (Delhaye, Jarocka, Barrea, et al., 2021). Neural responses do not encode the magnitude of friction itself; rather, they reflect the progression of local slip within the contact area, making humans highly sensitive to slip events. In addition, spike rate correlates with the magnitude of sliding friction, while transient friction changes elicit single, time-locked spikes whose latency reflects the sharpness of the frictional change (Dione, Watkins, Vezzoli, et al., 2021).

Only a few studies have directly addressed the resulting perception of friction and investigated how well participants can judge friction differences. Following Weber’s law, the perception of friction is expected to reflect relative rather than absolute differences (Gueorguiev, Vezzoli, Mouraux, et al., 2017). Humans can reliably discriminate smooth materials based on friction alone, and subjective slipperiness ratings closely follow the physical coefficient of friction (Smith and Scott, 1996). Friction differences between samples with the same material but different surface textures are perceived when the difference exceeds 15-20 % (Fehlberg, Kim, Drewing, et al., 2022). Humans are highly sensitive to transient friction changes, with a just-noticeable difference (JND) of about 11 % for ultrasonic friction modulation displays (Gueorguiev et al., 2017).

### Entanglement of roughness and friction perception

Although the five tactile dimensions represent distinct perceptual constructs, experimental evidence shows that they are not orthogonal but exhibit interactions (Janko, Wiertlewski, & Visell, 2018; Okamoto et al., 2013; Smith et al., 2002; Tan, Choo, Lin, et al., 2022; Skedung, Harris, Collier, et al., 2020). For example, the rate of change in tangential force is a significant determinant of roughness perception (Smith et al., 2002). To add to the complexity, the measured friction is a non-monotonic function of surface roughness. Friction is high for smooth surfaces and initially decreases with increasing surface roughness due to a reduction of the adhesive contact area, but rises again above a threshold when dynamic skin deformation by surface asperities increases dissipation (Derler and Gerhardt, 2012; Saikumar et al., 2025).

Katz’s duplex theory proposes two perceptual pathways for texture processing, and recent work suggests that friction perception should be investigated in the light of this theory as well. One psychophysical study indicates that friction perception can be linked to the coarse texture channel, as skin stretch increases when moving across surfaces with higher friction (Skedung et al., 2020). In previous work, we found that friction perception differed for the only sample in the tested set whose texture asperities were smaller than 100 µm in all dimensions and proposed that the scale of surface texture interferes with the perception of friction (Fehlberg et al., 2022).

**Fig. 1:**
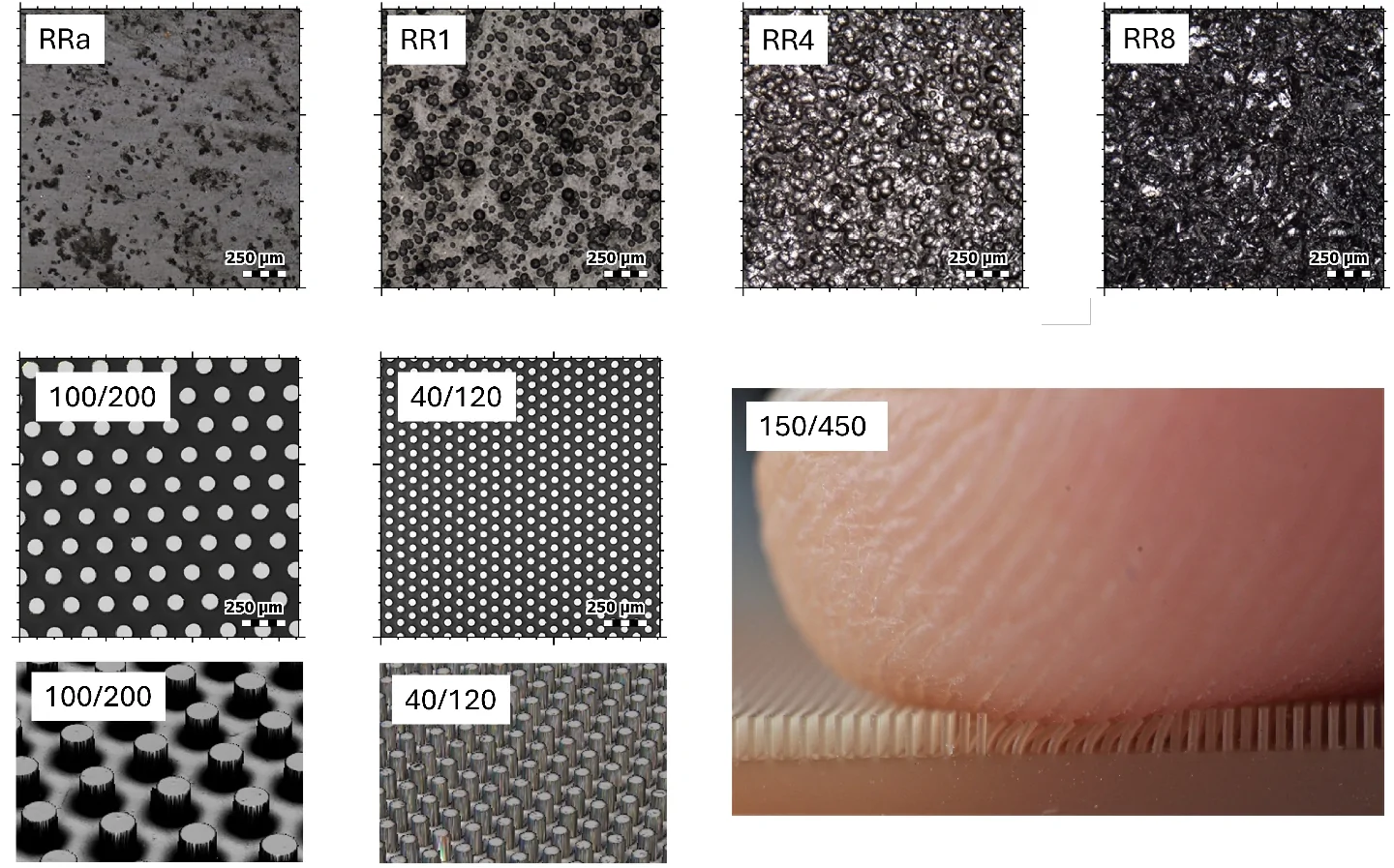
*Top*: Microscopy images of four representative randomly rough samples with increasing roughness from left to right. *Bottom left*: Confocal microscopy images of representative MS samples, with corresponding 3D surface topography. *Bottom right*: Photography of a finger sliding from left to right over the microstructured sample with pillar diameter of 150 µm and a height of 450 µm. The pillars in contact with the finger are bent under the normal and tangential forces

In this work, we present three experiments which aim to relate distinct aspects of tactile perception: roughness discrimination, texture detection, and friction discrimination. The experiments are connected by the set of stimuli, namely microstructured rubber surfaces with a periodic array of bendable micropillars of varying dimensions. This sample set offers a parameterized model system of soft surfaces with fibrillar structure. In the first experiment, we compare roughness perception by sample sorting and insertion tasks. We correlate the sorting results with physical parameters that describe randomly rough surfaces and surfaces with periodic microstructures. We derive a metric of perceived roughness for the microstructured textures, which cannot be derived from established geometrical definitions of statistical roughness. In the second experiment, we explore the perceptual detection of microtextures, with a focus on differences between static and dynamic touch. We investigate whether randomly rough and microstructured samples are perceived similarly as being textured, or whether the random versus periodic structures lead to different texture judgments. In the third experiment, we build on the results of the first two experiments to test the hypothesis that friction perception follows the duplex theory, and thus differs between surfaces with microstructure dimensions above and below a threshold of ≈100 µm.

## Samples

Two sets of rubber samples were prepared for the psychophysical experiments. The first set consisted of 13 samples with randomly rough surfaces, and the second of eight microstructured samples with periodic arrays of micropillars. Both sample sets were produced using the replica molding technique with negative molds made of the polydimethylsiloxane (PDMS) Elastosil M4601 (Wacker Chemie AG, München, Germany) and positive samples of the polyurethane (PU) PMC770 (Smooth-On, Inc., PA, USA). The randomly rough samples were replicated from aluminum samples with varying roughness created by sandblasting with different sand-particle sizes and blasting durations. These samples will hereafter be referred to as randomly rough (RR) samples. The microstructured samples were replicated from microfabricated hexagonal arrays of silicon pillars (Institute of Semiconductors and Microsystems, TU Dresden, Germany). The pillar dimensions on the eight samples were diameter[µm]/height[µm] 40/80, 40/120, 60/120, 60/180, 100/200, 100/300, 150/350 and 150/450 (Fig. 1). With this size selection, we aimed to cover texture scales below and above the transition region proposed by the duplex theory. The distance between pillars was equal to their diameter, so that the flat top areas of all micropillars covered 22.67 % of the surface for all samples. These eight samples will hereafter be referred to as microstructured (MS) samples. All 21 samples were 50 mm x 50 mm. Dynamic mechanical analysis (DMA) of the bulk PU material resulted in an elastic modulus of 4.5 MPa.

Surface roughness is often described following the standards in ASME B46.1 by the root-mean-square variation of the height profile

1 $$\begin{aligned} R_q&= \sqrt{ \frac{1}{L} \int _0^L z^2(x) ~ dx } \end{aligned}$$

with *L* the length of a height profile and *z*(*x*) the deviation from the mean height, or by the mean peak-to-valley height

2 $$\begin{aligned} R_z&= \frac{1}{N} \sum _{i=1}^{N} (z_{\text {max},i} - z_{\text {min},i}) \end{aligned}$$

with *N* the number of adjacent local maxima $z_{\text {max},i}$ and minima $z_{\text {min},i}$, or by the root-mean-square slope of the surface topography

3 $$\begin{aligned} R_{\Delta q}&= \sqrt{ \frac{1}{L} \int _0^L \left( \frac{dz(x)}{dx} \right) ^2 dx } \end{aligned}$$

where *dz*(*x*)/*dx* is the local slope of the height profile.

The roughness parameters $R_q$, $R_z$ and $R_{\Delta q}$ capture different but complementary physical aspects of surface texture. $R_q$ represents the statistical height variance of the profile, describing how widely the profile heights are spread around the mean line. $R_z$ highlights individual peaks in valleys on macro scale and is therefore sensitive to prominent asperities. $R_{\Delta q}$ quantifies the mean steepness of the surface profile.

The 13 randomly rough rubber samples had a measured $R_q$ ranging from 0.04 to 7.80 µm, $R_{z}$ from 0.23 to 39.27 µm, and $R_{\Delta q}$ from 0.037 to 0.302. These roughness values are averages calculated from 30 profilometer scans (Surfcom 1500SD3, Accretech GmbH, Germany) with tip size, wavelength, and cut-off filter chosen according to DIN EN ISO 4288:1998 and 3274:1998. One drop of rapeseed oil was used as a lubricant to prevent stick-slip between the samples and the profilometer tip. The roughness values for all 13 samples are summarized in Table 1. For our sample set, these values of the different roughness parameters are highly correlated (0.97<r<1), although the roughness ranks do not match for all samples and parameters. Comparing $R_{q}$ and $R_{z}$, we observe a different order for samples RR1 and RR2 and for samples RR6 to RR8. Comparing with $R_{\Delta q}$, the ranks of RRc and RR4 are exchanged.

**Table 1:** Measured surface roughness of the randomly rough rubber samples

sample	$R_{q}$ [µm]	$R_{z}$ [µm]	$R_{\Delta q}$
RRa	0.042±0.039	0.229±0.230	0.037±0.022
RR1	1.130±0.270	5.963±1.543	0.090±0.022
RR2	1.323±0.619	5.954±2.531	0.077±0.037
RRb	1.752±0.346	8.146±1.613	0.126±0.038
RR3	3.110±0.312	15.633±1.823	0.130±0.016
RRc	3.592±0.418	17.962±2.097	0.162±0.029
RR4	3.677±0.352	18.611±2.204	0.155±0.023
RR5	4.839±0.683	25.683±3.777	0.237±0.04
RRd	4.913±0.649	27.498±3.536	0.258±0.047
RR6	5.551±0.419	32.379±3.201	0.273±0.021
RR7	5.657±0.712	31.097±3.185	0.267±0.040
RR8	6.041±0.795	31.238±3.750	0.273±0.023
RRe	7.801±0.812	39.272±4.379	0.302±0.042

For the periodic topography of the microstructured samples with infinitely steep slopes, the roughness measures defined above fail to represent the surface structure. $R_q$ and $R_z$ depend on the pillar height only, but not on the pillar diameter, and $R_{\Delta q}$ of these samples cannot be determined due to the infinite slope of the pillar walls. We will explore a scale of perceived roughness upon tactile exploration in this study.

We conducted three experiments with focus on roughness (Experiment I), texture (Experiment II), and friction perception (Experiment III). Experiment I includes all 21 samples, Experiment II includes all MS samples and a selection of five RR samples (RRa - RRe), whereas Experiment III focuses on the eight MS samples. Experiment I and II were conducted with the same participants on the same day. Experiment III was conducted 5 months before Experiments I and II. The long break between experiments minimizes a possible bias in the participants’ response to the roughness and texture tasks (Experiments I and II) based on the previous friction perception (Experiment III).

## Experiment I: Roughness perception

Roughness judgments were obtained from 30 participants (18 female, two left-handed, aged between 21 and 38, average age: 28.0±3.9). This sample size is comparable to or exceeds those used in related psychophysical studies of roughness perception (Bergmann Tiest and Kappers, 2007; Hollins and Risner, 2000; Skedung et al., 2011) and was sufficient to reliably detect the observed effects. This experiment consisted of three tasks, two tasks of *sorting* microstructured and randomly rough samples (Exp. Ia, Ib), and one task of *inserting* additional samples into the previously established scale of randomly rough samples (Exp. Ic).

**Fig. 2:**
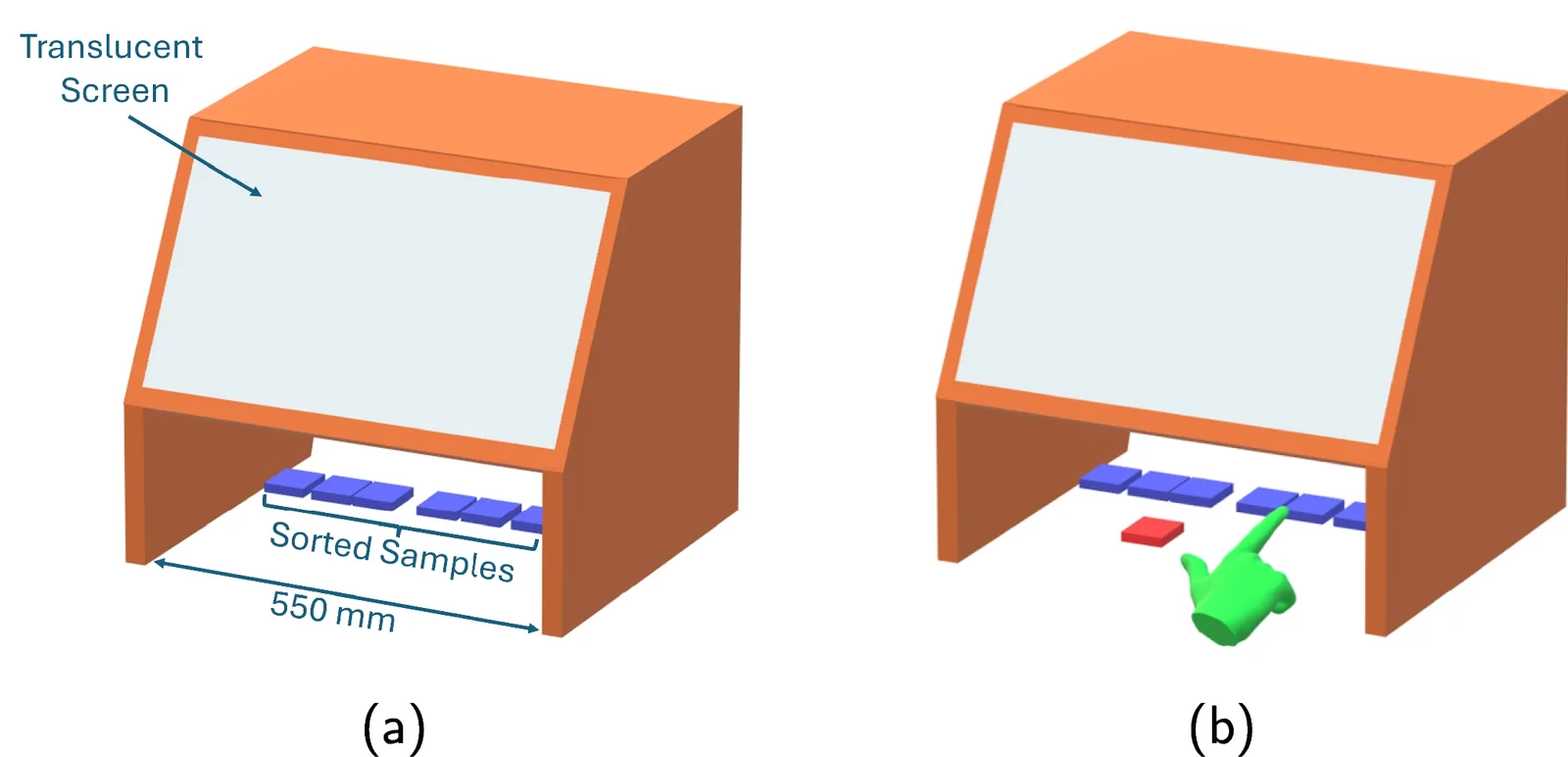
Experimental setup used in Experiment I. The translucent screen allowed the participants to see the location of the samples but not to recognize their texture. (**a**) Exp. Ia&b: The samples were sorted inside the box with a width of 550 mm. (**b**) Exp. Ic: The participants were asked to explore additional samples (*red*) and to then insert them into the scale of randomly rough samples by pointing to the position they would choose

In Exp. Ia, participants were asked to sort the microstructured samples based on their perceived roughness. They explored the eight samples using circular motions with their dominant index finger, sorting them from least rough on the left to most rough on the right. The samples were to be placed on a linear scale of 550 mm, with the distances between them representing the perceived differences in roughness. The participants were asked to place the least and most rough samples at the edges of the box shown in Fig. 2a

The position of the center of the sorted samples was marked on a paper strip and measured with a ruler, rounded to 5 mm precision and then normalized to a scale between 0 and 1 with 1 being the sample that was indicated as most rough. Participants generally arranged the samples in a straight line along the scale. If a sample was placed slightly diagonally, the horizontal position of its center was used for the analysis. We refer to this normalized subjective ordering as the perceived roughness scale (PRS). For Exp Ib, the microstructured samples were removed and participants were asked to sort a selection of eight randomly rough samples (RR1-RR8) with respect to perceived roughness, with the same procedure as in Exp. Ia.

With the established scale of perceived roughness for the RR-samples, we conducted the insertion task of Exp. Ic for all eight microstructured samples and five randomly rough samples (RRa-RRe). Participants were asked to explore the samples one after the other and to point to the place in the roughness scale at which they would insert the sample based on perceived roughness. The randomly rough samples on the scale were repeatedly explored to find the position to insert the presented sample. After each decision, we removed the presented sample and provided the next one, so that the participants always inserted one sample into the scale of eight randomly rough samples. A schematic image of this procedure is shown in Fig. 2b. The order of samples to insert was randomized for all participants. The participants were not restricted in time. They needed between 8 and 20 min to sort each sample set and around 10 to 20 min to insert the additional samples.

At the end of the experiment, we measured the skin moisture of the dominant index finger (Corneometer, Courage+Khazaka electronic GmbH, Germany).

### Results of Experiment I

#### Exp. Ia & Ib

The participants strongly agreed on the perceived roughness for the randomly rough samples, with a mean Spearman rank correlation of 0.88. The agreement for the microstructured samples is even stronger, with 0.92 (excluding ID13 and ID20). Participants ID13 and ID20 sorted both sample sets in disagreement with those of all other participants and are therefore excluded from all following analyses. A correlation matrix is provided in Fig. S1 in the Supplementary Information.

The average Spearman rank correlation between the physical roughness parameters (Table 1) and the individual PRS is 0.85, 0.92, and 0.87 for $R_{q}$, $R_{z}$, and $R_{\Delta q}$. The average agreement among participants with respect to the PRS is thus better than or equal to the average agreement with the physical roughness measurements $R_{q}$ and $R_{\Delta q}$.

**Fig. 3:**
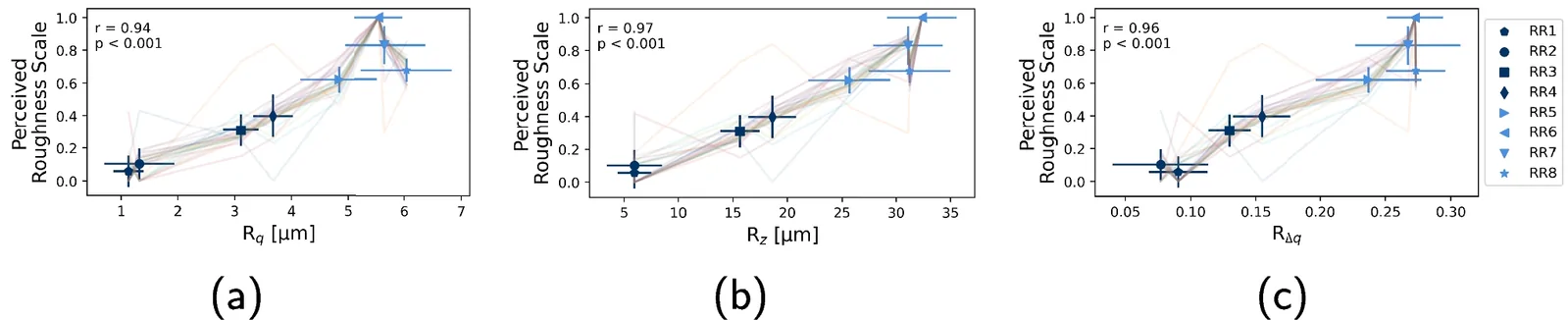
Perceived roughness scales (PRS) as a function of the measured roughness parameters $R_{q}$, $R_{z}$ and $R_{\Delta q}$. *Dots* and *error bars* indicate the average PRS with standard deviations. The Pearson correlations are reported within the figures. The *dark* and *light blue dot colors* indicate two clusters of samples identified by applying the *k*-means algorithm to the distance matrices of the participants’ perception scales (see text). *Light colored lines* report the individual PRS

By taking the distances between the sorted samples into account, we calculated Pearson correlations to quantify the agreement between perceived and measured roughness distances. The measured roughness parameters are highly correlated with the average PRS of the participants, with Pearson correlations of 0.94, 0.97, and 0.96 for $R_q$, $R_z$, and $R_{\Delta q}$, respectively (see Fig. 3).

**Fig. 4:**
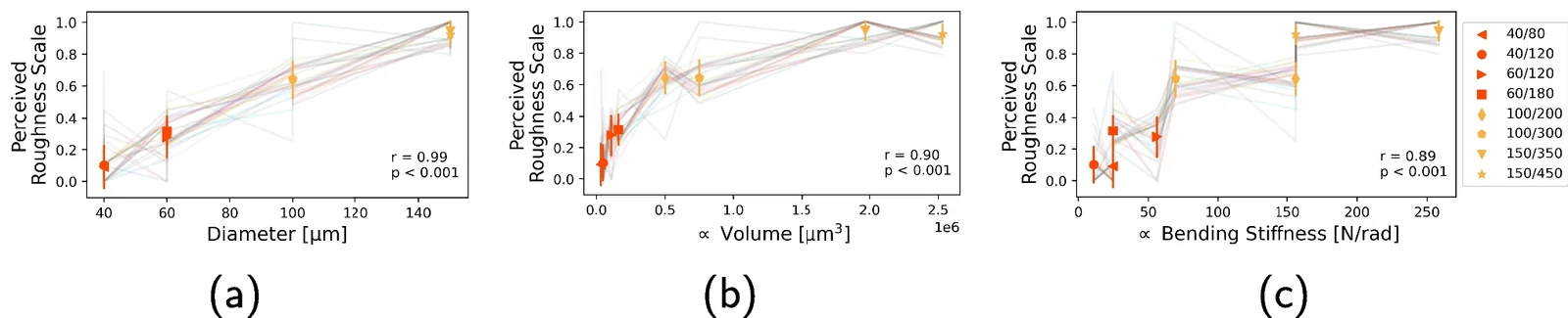
Perceived roughness scale for the microstructured samples as a function of (**a**) the pillar diameter, (**b**) the pillar volume, and (**c**) the bending stiffness. *Dots* and* error bars* indicate average PRS and its standard deviation. The *dark* and *light orange dots* represent two sample clusters that were identified by applying a *k*-means algorithm to the participants’ perception (see text). The *light colored lines* indicate the individual perceived roughness scales

The individually perceived roughness scales are compared with the measured roughness values through light colored lines in Fig. 3. We found significant Pearson correlations (0.78<r<0.98,p<0.022) for the three roughness parameters for all individuals but two. While the PRS of ID11 was significantly correlated with $R_{z}$ and $R_{\Delta q}$, the correlation with $R_{q}$ was just not significant (r=0.7 and p=0.052). The PRS of ID12 was not significantly correlated with either of the three parameters (r<0.56,p>0.15). We calculated the average of all Pearson correlations of the individual PRS with the measured roughness values using Fisher-*Z*-transformation. We found a strong average correlation of 0.92, 0.95, and 0.94 for $R_{q}$, $R_{z}$, and $R_{\Delta q}$, respectively (p<0.001).

Figure 3 shows that the sorting correlation depends on which physical roughness parameter is used as ground truth. For $R_q$, systematic deviations occurred primarily in the three roughest samples, resulting in lower Spearman and Pearson correlations. $R_z$ and $R_{\Delta q}$ showed deviations only between two roughest samples, but additionally also for the two smoothest samples. However, the large standard deviations in the measured roughness values limit the interpretation of these relationships and prevent a clear prioritization of physical parameters in the prediction of human sorting.

For the microstructured samples, we investigated how the diameter and the height of the pillars influenced the perception of roughness. Figure 4 shows the average perceived roughness scale as a function of the pillar diameter *D*, the pillar volume ($\propto ~D^2\cdot H$, with *H* being the pillar height), and the bending stiffness of the pillars ($\propto ~D^4/H^2$). All three combinations were significantly correlated with the average PRS.

The pillar diameter explains 98 % of the PRS ($R^2_{\text {adj.}}$ = 0.98), which underlines that the perceived roughness of MS samples is primarily determined by the pillar diameter. Adding the pillar height to the linear model did not increase the explained adjusted variance (D+H: $R^2_{\text {adj.}}~=~0.98$). The lack of influence of the pillar height on the perceived roughness can also be derived from individual answers when comparing the perceived roughness position of samples with the same pillar diameter but different height: In 53 of 112 cases, the sample with lower pillar height was judged to be rougher, which is close to chance level.

The light lines in Fig. 4 represent the individual perceived roughness scales of the participants. We find strong average Pearson correlations of 0.96 for pillar diameter, of 0.86 for the pillar volume, and 0.87 for the bending stiffness. This average of the correlations for all 28 participants was calculated using Fisher-Z transformation. For this sample set, we identify two PRS that are not significantly correlated with any of the three pillar parameters. The sorting by ID08 showed no significant correlation with the bending stiffness (r=0.46,p=0.26), and by ID11 with none of the three parameters (r<0.68,p>0.06).

**Fig. 5:**
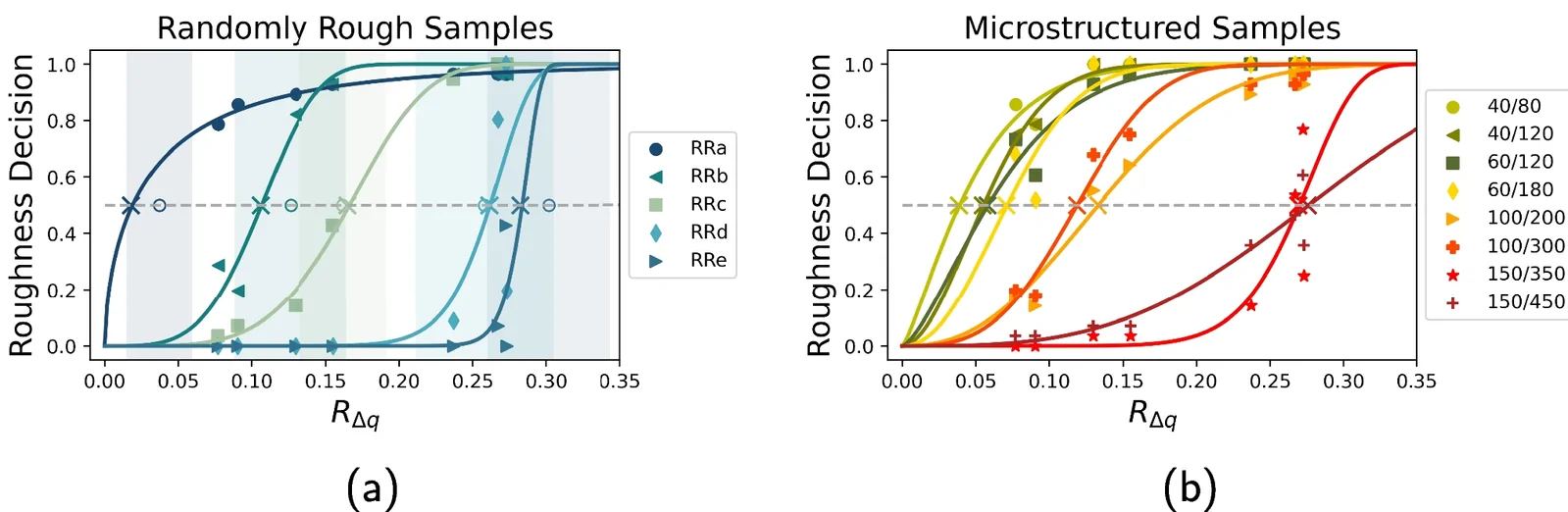
Average roughness decisions derived from the insertion task in Exp. Ic. (**a**) Results for the five control RR samples. *Hollow dots* represent the measured $R_{\Delta q}$ for the five control samples, the *colored sections* represent the standard deviation for this measurement. (**b**) Results for the microstructured samples. In both figures the *crosses* mark the point of subjective equality

As the microstructured samples were selected to cover different texture scales, we additionally explored whether this design-based grouping is reflected in the perceptual similarity data. We therefore calculated distance matrices from the individual sample distances in the PRS for all participants and applied a cluster analysis. Silhouette analysis indicated an optimal solution of k = 2 for both sample sets (MS: silhouette = 0.497, RR: silhouette = 0.429). Cluster significance was verified using a permutation test with 1000 iterations, in which the sample labels were randomly shuffled before recalculating the within-cluster sum of squares (WCSS). Both cluster structures were highly significant (WCSS; MS = 9.71, p<0.001; RR = 9.90, p<0.001). The k-means model (k = 2) was then used to assign samples to the two clusters. For the MS dataset, the samples formed two clusters, one containing samples 40/80, 40/120, 60/120, and 60/180, and the other containing 100/200, 100/300, 150/350, and 150/450. The RR dataset was similarly separated into two clusters, comprising samples RR1–RR4 and RR5–RR8, respectively. The identified clusters reflected the presentation of two ranges of structural dimensions, suggesting that this distinction was also captured in the participants’ roughness judgments. To visualize the identified sample clusters, we chose cluster-based colors in Figs. 3 and 4.

The definition of a roughness scale based on the sample positions may be affected by the sample size and the limited space for placing the samples. The size of the samples introduces a minimum distance in the perceived roughness scale, which may be larger than intended by participants. We have therefore repeated the analysis based on the gaps between sorted samples, focusing more on variations in the perceived difference between adjacent samples. The linear models and clustering have the same overall results with minor quantitative differences (see SI section *Influence of sample dimensions* and Fig. S2 and S3 for details).

#### Exp. Ic

The perceived roughness scales for microstructured samples clearly reflect the different pillar diameters (see Fig. 4a), while the physical roughness parameters $R_q$ and $R_z$ introduced above reflect the pillar height only. To establish a metric for the perceived roughness in comparison to RR samples, we analyzed the results of the *insertion* task in Exp. Ic.

The insertion task started from the roughness scale established with the eight randomly rough reference samples (RR1–RR8) in Experiment Ib. Participants could repeatedly explore the reference samples while performing the task. For each trial, one additional sample (either an MS sample or one of the control samples RRa–RRe) was presented. Participants explored the sample and indicated where they would place it within the existing roughness scale based on its perceived roughness. After the decision, the sample was removed and the next sample was presented. Each of the 8 MS and 5 RR samples was inserted once by each participant. To construct psychometric curves and extract points of subjective equality (PSEs), we analyzed the insertion decisions across all participants. For each inserted sample, we compared its insertion position with the position of each RR reference sample in the roughness scale.

We assigned a comparison value to each pair consisting of an inserted sample and one of the RR reference sample in the scale. A value of 1 was assigned if the inserted sample was perceived as less rough, 0.5 if it was perceived as equally rough, and 0 if it was perceived as rougher than the respective RR sample from the reference scale. The comparison values were then averaged across all participants for each inserted sample and RR reference sample combination.

The average decision value for each inserted sample in the RR scale is plotted as function of the respective $R_{\Delta q}$ for the five RR samples in Fig. 5a and for the eight MS samples in Fig. 5b

The validation with the five RR samples demonstrates that the insertion task is suitable to establish a metric for perceived roughness in terms of physical roughness parameters of randomly rough surfaces. The PSEs for the three samples RRb, RRc and RRd fall into the standard deviation of the measured values for $R_{q}$, $R_{z}$ and $R_{\Delta q}$. Please note that RRa and RRe have roughness values outside the scale of RR samples and, therefore, were mostly perceived as less rough (RRa) or rougher (RRe) than the extremes of the roughness scale. This configuration limits the precision of the PSEs derived from the fit beyond its support points. The derived values for the perceived roughness are highly correlated with the measured roughness of the five control samples ($R_{q}$ and $R_{\Delta q}$: r=0.99,p<0.002; $R_{z}$: r=0.97,p=0.007).

The extracted PSE values for the microstructured samples ($R_{q, \text {perc.}}$, $R_{z, \text {perc.}}$ and $R_{\Delta q, \text {perc.}}$) are summarized in Table 2. The PSEs based on all three roughness parameters are correlated with the average PRS of Experiment Ib (r>0.93, p<0.001) and with the pillar diameter (r=0.98 for $R_{q, \text {perc.}}$ and $R_{\Delta q, \text {perc.}}$, and r=0.96 for $R_{z, \text {perc.}}$, p<0.001).

**Table 2:** Perceived surface roughness ($R_{z}$, $R_{q}$ and $R_{\Delta q}$ for five RR samples and eight MS samples)

sample	perceived $R_{q}$ [µm]	perceived $R_{z}$ [µm]	perceived $R_{\Delta q}$
RRa	0.12	0.576	0.018
RRb	1.944	9.634	0.106
RRc	3.825	19.54	0.165
RRd	5.433	31.126	0.261
RRe	10.154	32.47	0.282
40/80	0.656	3.065	0.038
40/120	0.576	5.897	0.055
60/120	0.784	3.657	0.058
60/180	1.104	5.169	0.071
100/200	2.793	14.067	0.133
100/300	2.408	12.018	0.119
150/350	5.897	31.526	0.269
150/450	5.937	31.894	0.276

The perceived roughness values for $R_{\Delta q}$ and $R_z$ are much smaller than the geometry of the samples would suggest. For example, the mathematical definition of $R_z$ would be equal to the pillar height, between 11 and 32 times larger than the value derived from the insertion task. We propose that the skin in contact with the pillar array does not sense the depth of the gap between pillars, and that the roughness perception is rather influenced by the bending of pillars under sliding touch (see Fig. 1).

### Discussion of Experiment I

The sorting results of the samples demonstrate a high level of agreement among participants in their roughness perception for both RR and MS samples. The consistency in the sorting of RR samples between participants was even higher than the consistency with the measured roughness parameters. For several sample pairs, the difference in measured roughness is much smaller than the respective standard deviations, leaving some uncertainty regarding the true order in roughness. This observation is in agreement with current discussions about the challenges of roughness measurement (Pradhan, Müser, Miller, et al., 2025). The roughness order given by the participants’ consistent sorting may actually be more reliable than the physical measurements.

One possible explanation is that the finger explores a much larger surface area and therefore gathers more information about the texture than the micro-meter probe in the line scans of the profilometer. Even with as many as 30 scans, the device still captures much less surface area than a single touch with a finger. As a result, human perception might produce a more stable and representative roughness ranking than the profilometer. Nevertheless, all three parameters $R_q$, $R_z$, and $R_{\Delta q}$ are significant predictors for the sorting of the RR samples by participants.

Our findings confirm previous reports that perceived roughness correlates with measured roughness parameters. The inter-participant correlations observed in our study were stronger than those found previously. One possible explanation is that our task (sorting eight samples of the same material) was less demanding than sorting 96 samples of different materials with a wider range of roughness, as in the study by Bergmann Tiest and Kappers (2007). The roughness range in a study by Skedung, Danerlöv, Olofsson, et al. (2011) was comparable to ours, and the lower inter-participant correlations in their study may be related to differences in the experimental design. In our experiment, participants were allowed to touch all samples during the sorting process, whereas Skedung et al. (2011) employed magnitude estimation to find a roughness order, requiring participants to remember previous samples.

**Fig. 6:**
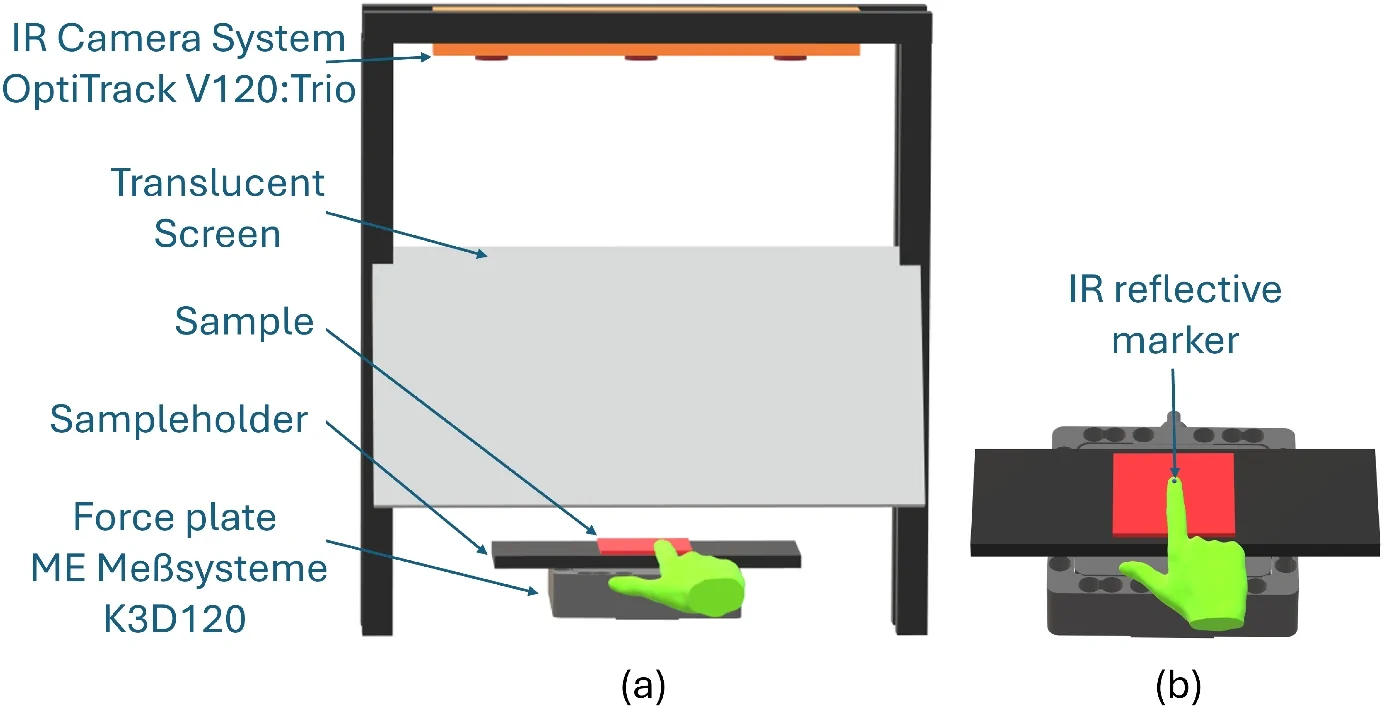
(**a**) Experimental setup for Experiments II and III. (**b**) A sample holder mounted on the force plate carries the samples during exploration. The IR-reflective marker is attached to the fingernail of the participant’s dominant hand

For MS samples, pillar diameter is the dominant factor, explaining 98 % of the variance in perceived roughness, while pillar height did not add to the explained variance. Pillar volume and bending stiffness did correlate with the perceived roughness, as expected, as they both scale with the pillar diameter. In our sample set, the pillar distance varied proportional to the pillar diameter, such that samples with larger pillar distance were perceived as rougher, consistent with previous findings on 3D-printed surface by Tymms, Zorin, and Gardner (2018). Since we varied pillar diameter and distance simultaneously to keep the relative fraction of exposed pillar surface constant, it remains unclear whether diameter or distance have the stronger influence on roughness perception.

The observed relationship between roughness perception and bending stiffness of pillars highlights the role of elasticity of the surface textures. Unlike the rigid microstructures used by Tymms et al. (2018), our pillars bend under sliding touch (see Fig. 1). This deformation increases the contact area between the finger and the surface (Fehlberg et al., 2022), with the largest contact area occurring at samples with lowest bending stiffness. These samples were rated as the smoothest in our experiment, aligning with the hypothesis of Tymms et al. (2018) that larger contact area contributes to the perception of a smoother surface. The strong correlation between bending stiffness and the roughness perception also aligns with the finding by Gedsun, Sahli, Meng, et al (2022) that bending stiffness is key parameter in the tactile perception of similarity between microfibrillar elastomer samples.

The MS samples with pillar diameters of 40 µm and 60 µm and heights between 80 µm and 180 µm form one cluster, whereas samples with pillar diameters of 100 µm and 150 µm and a height ranging from 200 µm to 450 µm form the second cluster with respect to similarly perceived roughness. The threshold of 100 µm suggested by Hollins and Risner (2000) for a separation of regimes in the duplex theory of tactile perception aligns with our separation of clusters by pillar diameter.

**Fig. 7:**
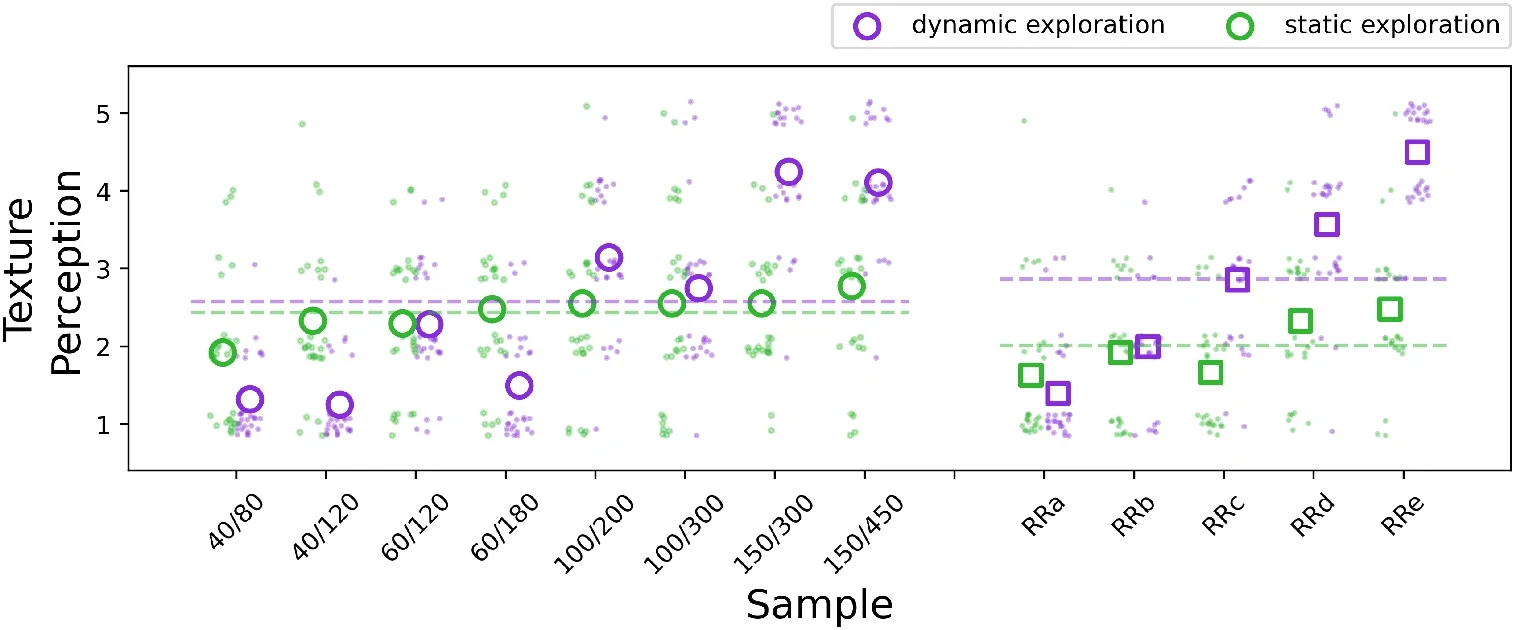
The rating of the perceived texture with 1: “The surface feels smooth”, and 5: “Clear perception of texture” is plotted for each participant (*small dots*) for both exploration procedures (dynamic (*purple*) and static (*green*)). The average answer is indicated by big hollow markers, for the MS samples with ∘ and for the RR samples with □. The average rating for each procedure and sample set is marked with a *dashed line*. The RR samples are plotted in order of increasing roughness

The points of subjective roughness equivalence for each of the MS samples, extracted from the insertion task, significantly correlate with participants’ perceived roughness scale, extracted from sorting. We conclude that inserting microstructured samples into a range of randomly rough surfaces is an effective method of establishing a perceptual roughness scale for samples lacking a mathematically defined roughness.

To summarize Experiment I, we find that participants are very consistent in rating perceived roughness of randomly rough as well as periodically microstructured surfaces. For the microstructured surfaces, the diameter of the bendable micropillars is the key parameter to predict perceived roughness. The roughness PSE of microstructured samples with respect to randomly rough samples is always much smaller than the pillar dimensions, indicating that only the contact with the edges of bent micropillars contributes to perceived roughness. The eight microstructured samples fall into two groups with similarly perceived roughness, four with diameter lower than 100 µm and four with diameter of 100 µm and larger.

## Experiment II: Texture perception

The tactility of surface textures was rated for static and dynamic touch by the same participants as in Experiment I. The sample set included the eight microstructured samples and five randomly rough samples (RRa-RRe) for comparison. The samples were located on a 3-axis-force sensor (K3D120 with GSV-8 amplifier, ME-Meßsysteme, Germany). The exploratory motion of the participants’ fingertip was captured by an IR-camera system (V120:Trio, OptiTrack, USA) with a reflective marker on the participants’ fingernail. A translucent screen blocked the sight so that participants were able to see the location of the sample but not their surface structure. A schematic image of the setup is shown in Fig. 6.

In a first task, participants were instructed to explore the samples three times with the index finger by static touch without performing lateral movements and to answer the question: “How different does this surface feel compared to a smooth surface?” Responses were given on a scale from 1 to 5, where 1 indicated that “the surface feels like a smooth surface” and 5 that they “clearly feel a texture”. We explicitly used ’textured’ rather than ’rough’ to investigate whether participants distinguish between the two terms. In a second task, participants explored the samples with three circular motions of sliding touch on each surface and answered the same question.

The presentation order of the samples was randomized in both tasks. To achieve comparable exploration procedures between participants we provided visual feedback of the applied normal force in real time and asked the participants to use 1.2 to 1.3 N for the static and 0.5 to 0.7 N for the dynamic exploration. To train the two exploration procedures, we first presented a paper sample on a stiff substrate, on which participants tested the exploration procedures as long as they wanted.

### Results of Experiment II

For the static exploration procedure, participants spent a total of 4.73±2.11 s in contact with the sample across the three separate touches. Participant ID11 on average pressed 75 % higher than instructed, so we removed their data from the analysis of the static exploration. The other participants used on average 1.34±0.15 N. In the dynamic exploration, all participants explored the samples within the target range with an average normal force of 0.50±0.10 N and touched the samples in total for 7.15±3.28 s.

We will first report the consistency in rating between participants. The average of the Spearman rank correlations is weak (ρ=0.1) for static and strong (ρ=0.75) for dynamic exploration (without ID13 and ID20). The correlation matrix is shown in the SI (Fig. S6).

In Fig. 7 we plot the individual answers of the participants for both exploration strategies and both sample sets. The responses for both sample sets span the entire range of available answer options, confirming that the tactility of both sample sets is comparable, and that the participants did not separate the rating scale between MS and RR samples. Despite the individual differences in ratings, we found trends for increasing texture perception with increasing roughness or pillar dimensions as shown in Fig. 7. For RR samples, the Fisher-Z-averaged correlations between measured roughness and the texture ratings after static exploration are moderate, with 0.46, 0.48, and 0.51 for $R_q$ (statistical height variance), $R_z$ (individual peaks and valleys) and $R_{\Delta q}$ (steepness of surface profile, p<0.001). After dynamic exploration, the average correlations are strong with 0.93, 0.93 and 0.92 (p<0.001) respectively, and the average rating increases monotonically with the measured roughness.

For the MS samples, the Fisher-Z-average correlation between the texture ratings and the pillar dimensions diameter, height, volume, and bending stiffness is weak for static exploration with 0.16<r<0.24 and strong for dynamic exploration with r>0.8. Interestingly, for dynamic exploration the diameter is again the best predictor with a strong correlation of r=0.90, while for the static exploration we find the height to be the best predictor with a weak correlation of r=0.24. This difference is also reflected in Fig. 7 where the ratings for the MS samples increase monotonically with increasing pillar diameter (not for height and volume) after dynamic exploration, while for static exploration the increase in texture rating with height is monotonic (not for diameter).

Analyzing the average of the answers for samples with the same pillar diameter, we found that participants tended to perceive less texture on samples with higher pillars during dynamic texture exploration. The opposite was observed for static exploration, where participants rated MS samples with the same diameter but higher pillars as more textured (see Fig. 7).

The contrasting effect of pillar height between static and dynamic conditions may reflect differences in mechanical interaction. Higher pillars bend more easily during dynamic exploration, reducing skin deformation and producing a smoother surface impression, whereas in static touch their increased compressibility may enhance texture perception when the finger penetrates the pillar array.

Static and dynamic exploration led to similar texture ratings for the MS samples, whereas the RR samples were rated lower after static and higher after dynamic exploration. This difference may be related to surface deformability. The bendable micropillars of the MS samples deform even under static contact, producing a perceivable compressive response. In contrast, the asperities of the RR samples deform less, resulting in smaller texture ratings after static exploration.

Texture ratings increased with structure size for both exploration strategies. However, the increase was much steeper during dynamic exploration. As a result, dynamic ratings were lower than static ratings for the four samples with the smallest pillar diameters (40 and 60 µm), but exceeded static ratings for the four samples with the largest pillar diameters (100 and 150 µm). This observation is in agreement with the two sample clusters for MS samples found in Experiment I.

### Discussion of Experiment II

Normal forces applied by participants were successfully constrained by visual feedback, in agreement with previous reports (Fehlberg et al., 2022; Papetti, Järveläinen, Giordano, et al., 2017). We can thus assume negligible differences in texture perception due to variations in this most relevant parameter of active exploration.

The participants rated the texture perception consistently after dynamic exploration, whereas their responses after static exploration were divergent. This difference between static and dynamic exploration reflects the critical role of temporal cues in the discrimination of fine textures (Hollins & Risner, 2000; Kwok, Darkins, Oddo, et al., 2010).

Hollins and Risner (2000) conducted a passive touch experiment using sandpaper samples with particle sizes between 9 and 350 µm. In a free magnitude estimation task, the participants were asked to rate the perceived roughness after static and dynamic passive touch. They found that static touch of samples with particle sizes below 100 µm results in an almost constant roughness estimate, while for larger particles the estimates scaled with the particle size. In contrast, the roughness estimates upon dynamic touch showed a consistent scaling with particle size across the full range. They concluded that temporal cues are required for perceiving the roughness of fine textures, whereas the roughness of coarse textures can be rated equally well through static and dynamic touch.

Hollins and Risner (2000) describe tactile perception in terms of particle size, such as the frequently cited 100 µm threshold for changes in perceptual cues. However, particle size does not directly correspond to standard surface roughness parameters used in materials science, nor to qualitative descriptors such as grainy, coarse, or smooth. This lack of equivalence makes direct comparisons across studies difficult, and the threshold cannot be easily transferred to research that relies on different measurement concepts or scales.

Similar to the results by Hollins and Risner (2000), we observe a crossover pattern between texture estimates in static and dynamic exploration. While both exploration strategies showed increasing texture ratings with structure size, static exploration yielded higher texture ratings than dynamic exploration, whereas the opposite was found for larger structures. Hollins and Risner (2000) show a similar crossover in their Fig. 1, where static exploration received higher roughness ratings for particle sizes below 30 µm. Above this size, the dynamic exploration resulted in higher roughness ratings. We propose that this crossover indicates a critical boundary in tactile texture perception. The sample clusters identified in Experiment I stand out in this analysis of the results, with samples of the first cluster below the crossover threshold and samples of the second cluster above.

Although it has been reported that lateral micro-slips may influence tactile perception even in static touch (Delhaye et al., 2021; Willemet, Kanzari, Monnoyer, et al., 2021), we believe that this mechanism can be excluded in our experiment. The combination of a high friction coefficient of rubber and of the surface compliance make micro-slips highly improbable.

To summarize Experiment II, we find that participants agree on texture ratings after dynamic more than after static exploration. Nevertheless both ratings are correlated with the pillar dimensions (MS samples) and the measured roughness (RR samples). The intersect between the texture rating of static and dynamic exploration splits the MS samples into the same groups that were found in the cluster analysis of Experiment I.

## Experiment III: Friction perception

The focus of this experiment was the perception of fingertip friction on MS samples. Specifically, we compared measured friction and the perceived friction. The motivation for this experiment was to test the duplex theory for structures of varying size, in a perceptual task which does not directly address the texture or roughness perception.

We recruited 41 participants who self-reported no neurological impairments. Of these, 23 also participated in Experiments I and II. This sample size is comparable to or exceeds those used in related psychophysical studies of friction perception (Gueorguiev et al., 2017; Smith and Scott, 1996) and was sufficient to reliably detect the effects observed in this study. To allow for direct comparison across studies, the same participant IDs were used.

The results of three participants (ID38, ID40, ID48) were excluded from the analysis. One participant damaged microstructures during exploration. The second participant misunderstood the task. The third participant gave inconsistent answers with a correct rate of significantly less than 50 % chance according to a binomial test. In the following, we thus analyze the results of 38 participants (22 females, one left-handed, average age: 29.3±6.1).

In this experiment participants explored the eight microstructured samples described before. Based on the two sample clusters found in Experiment I and the difference in texture perception for these two sample clusters in Experiment II, we group the samples and refer to the samples 40/80, 40/120, 60/120, and 60/180 as fine-textured surfaces (group A) and to the samples 100/200, 100/300, 150/350, and 150/450 as coarse-textured surfaces (group B). These groups therefore reflect the perceptual clustering observed in Experiments I and II rather than a classification based solely on physical dimensions.

The experiment consisted of two parts, separated by a short break to allow participants to rest. We selected 21 sample pairs: all six possible combinations within both groups A and B, and 9 out of the 16 possible inter-group comparisons. This reduction ensured a balanced number of comparisons across intra- and inter-group conditions. We excluded the samples with the second-largest structures from each group (60/120 and 150/350) from the inter-group comparisons.

In both parts of Experiment III, we asked the participants to explore the 21 sample pairs in circular motion with the straight index finger and to answer the forced-choice task: *“On which of the two samples is it more difficult to move the finger over the surface?”*. To answer this question, participants could touch the samples of each pair as long as they wanted and switch between the samples as often as they wanted. After each decision, the next sample pair was presented. During exploration, the samples were located behind a translucent screen, and the participants wore noise-reducing headphones to avoid visual or auditory feedback for their decision. The sample pairs were placed on a three-axis force sensor (K3D120 with GSV-8 amplifier, ME-Messysteme, Germany) to track the normal force ($F_N$) and the lateral forces ($F_x,F_y$) during the whole exploration. The motion of the participants’ finger during the exploration was captured by an Infrared Camera System (V120:Trio, OptiTrack) that tracks the position of an IR reflective marker placed on the fingernail. The setup was the same as in Experiment II (see Fig. 6). After the break, we repeated all 21 pairs so that all participants touched each pair twice. The order of sample pairs was randomized in both experimental parts.

Before and after the experiment, we measured the skin hydration of the dominant index finger (Corneometer, Courage+Khazaka electronic GmbH, Germany).

**Fig. 8:**
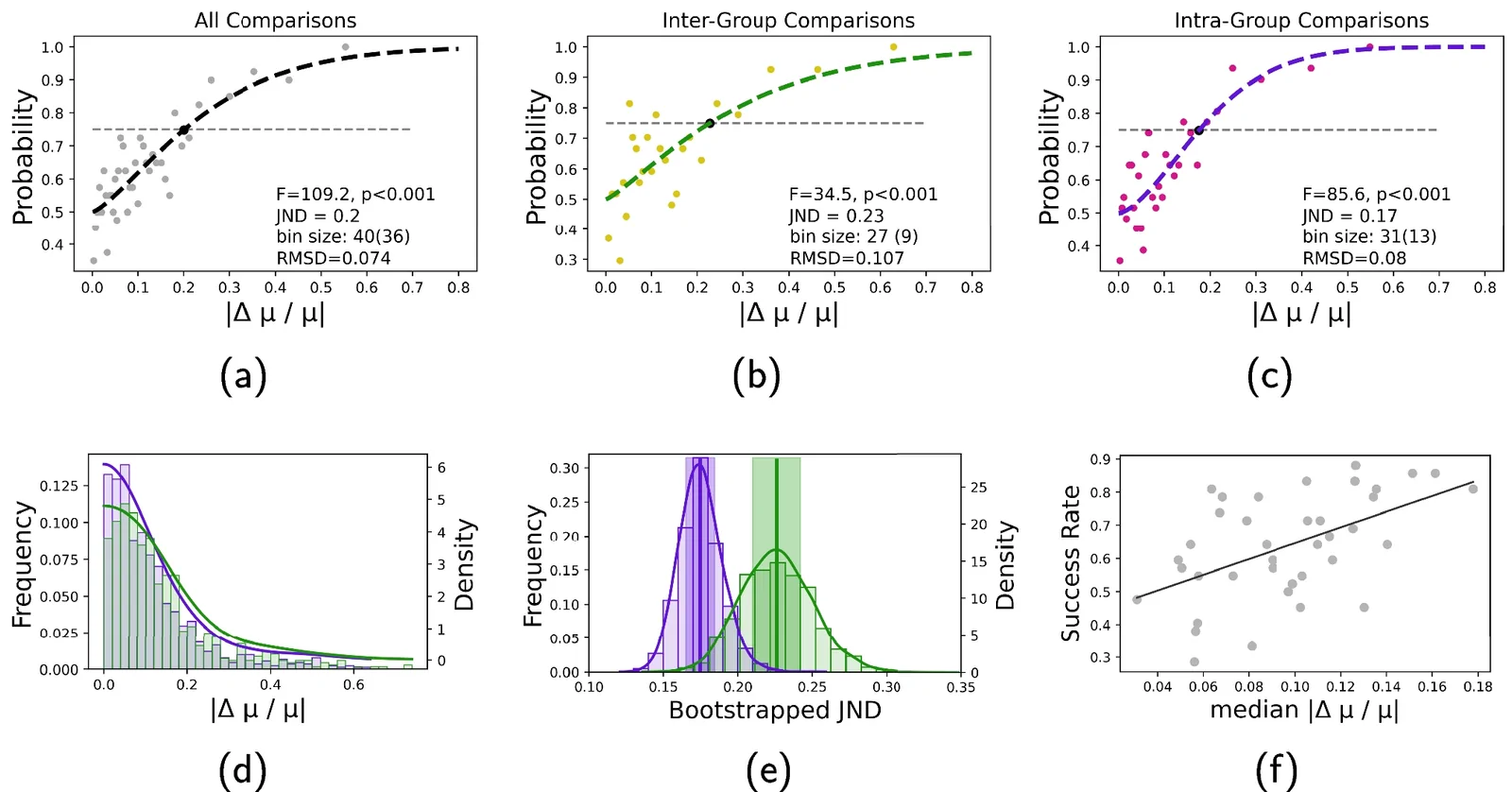
**a**)-**c**) The probability that participants answered in agreement with the measured coefficient of friction is plotted against the relative difference in friction coefficient $|\Delta \mu /\mu |$. a) Summary of all sample comparisons. **b**) Summary of comparisons between samples from different groups. **c**) Summary of the comparisons between samples within both groups. **d**) Histogram of the relative differences in measured µ for both inter- (*green*) and intra-comparisons (*purple*), with kernel-density estimation (KDE). **e**) Histograms of JNDs calculated from bootstrapped datasets for both inter- (*green*) and intra-group (*purple*) comparisons. The medians, IQS, and KDE are marked with the same color scheme. **f**) The individual success rate of the participants, including all sample pairs is plotted against the median of the relative difference in friction coefficient

### Data analysis of Experiment III

Before analyzing the force data, we removed 50 Hz noise by applying a moving-average filter over ten data points (≈83 ms). The friction force ($F_F$) was calculated as $F_F=\sqrt{F_x^2+F_y^2}$ and the coefficient of friction (μ) following Amontons’ law as $\mu =F_F/F_N$. We selected the data of interest as those with $F_F~>~0.05$ N and $F_N~>~0.05$ N. By setting this threshold, only the data points representing interaction between the fingertip and the sample were taken into account.

Position data were used to determine the radii chosen by individuals for circular exploration, as well as the speed (*v*) of finger movement in the x-y plane ($v=\sqrt{\Delta x^2 +\Delta y^2}/\Delta t$, with $\Delta t= 1/120~\text {s}$).

In the following, we report median friction force, median friction coefficient, and median velocity per sample exploration and per participant. Where we report mean values across trials, we calculate them using the mean of these medians. For comparison, we also normalize results by dividing the friction values of each exploration by the mean friction over all explorations of that participant. Normalized values allow for a relative comparison of friction between samples while avoiding a dominant contribution of participants with higher mean friction.

### Results of Experiment III

During the exploration of the samples, participants applied different normal forces, sliding speeds, and circle radii (*r*). These parameters are reported as median value for normal force and speed and as mean value for the circle radii, which was the only parameter normally distributed (Shapiro–Wilk test, W=0.98,p=0.63). The participants explored the samples with median speeds between 26.09 and 134.03 mm/s (median over all participants: 48.96 mm/s, IQR: 38.24-58.49 mm/s) and with an average circle radius of 10.82±2.45 mm. The median normal force of the participants was between 0.12 and 1.85 N (median over all participants: 0.48, IQR: 0.27-0.85). Exploration frequencies varied among sample pairs, with 1 to 10 touches per sample. The wide range of parameters describing the exploration procedures clearly shows that participants adopted very different approaches for sample exploration when left free to choose the normal force, speed, and duration of interaction. In addition to finger skin moisture, these differences in exploration strategies result in different friction coefficients measured across participants, as the measured average friction coefficient ranges between 1.46 and 2.78 (average over all participants: 2.18±0.29). As already mentioned, we report normalized friction coefficients to avoid greater influence of some participants on the correlations.

To examine the influence of surface structures on friction, we repeated the correlation analysis from the previous experiments using the normalized friction values. We found no correlation between the average normalized friction coefficient per sample and the pillar dimensions diameter (r=-0.49,p=0.22), height (r=-0.24,p=0.56) and volume (r=-0.25,p=0.56), and a just-not significant correlation with the bending stiffness of the pillars (r=-0.66,p=0.076, see SI Fig. S7).

The probability of participants ranking the perceived friction in agreement with the relative difference $\Delta \mu /\mu$ for the sample pairs is summarized in the group-based psychometric curves shown in Fig. 8. Including decisions of all participants for all pairs, we found a just noticeable difference (JND) in friction of 20 % (see Fig. 8a

We separated the trials into two categories: intra-group comparisons between samples within either group A or group B and inter-group comparisons between one sample from group A and one from group B. We found a higher JND of 23 % for inter-group comparisons and a lower JND of 17 % for intra-group comparisons (see Fig. 8c

As one can derive from the friction results in Fig. 8d, the two categories exhibit a substantial overlap in relative friction differences. This overlap suggests that the grouping was reasonably balanced with respect to friction, and that neither category was prone to be easier or harder. The decision accuracy is similar in both intra-group and inter-group comparisons with 63.9 % and 63.7 %, respectively, even though the median $\Delta \mu /\mu$ for inter-group pairs was slightly larger (10.2 %, IQR: 5 - 17.5 %) than for intra-group pairs (8.2 %, IQR: 3.9 - 15.1 %). We therefore consider it unlikely that the observed difference in JNDs is primarily explained by differences in the distribution of measured $\Delta \mu /\mu$.

To verify a difference between the JND values for inter- and intra-group comparisons, we calculated the JND distributions from 10,000 bootstrapped data collections for both categories and present the result in Fig. 8e. The median JNDs were found to be 22.6 % for inter-group comparisons (IQR: 21 - 24.1 %) and 17.5 % (IQR: 16.5 - 18.4 %) for intra-group comparisons, respectively. A Mann–Whitney *U* test was conducted to examine the differences between the distributions. The results confirm a significant difference between inter- and intra-group comparisons (U=96902842,p<0.001).

It was thus more difficult for participants to compare friction between samples with fine and samples with coarse texture than between pairs of samples of either fine or coarse texture. As reliable individual JND estimates could not be obtained for all participants, we focused on group-level JNDs. The bootstrap analysis provides an estimate of the variability of these JND values.

We also observed that participants had more difficulty detecting friction differences within group A than within group B (see SI S8). For intra-group-A comparisons, we determined a JND of 21 % and for intra-group-B comparisons a JND of 15 %.

Note that due to the small values of relative differences in the intra-group A comparisons, only two of the binned data points in Fig. S7a are greater than the calculated JND. We therefore calculated the JND for 10,000 bootstrapped samples of intra-group A and intra-group B comparisons (see histograms SI (S8c)). The medians of the calculated JND distributions are 20.7 % for the intra-group A (IQR: 19.2-22.3 %) and 14.7% for intra-group B (IQR: 13.6-15.9 %). Both distributions are significantly different from the bootstrapped distribution of the inter-group comparisons following the Mann–Whitney *U* test (group A: U=70611397, group B: U=99586208.5, p<0.001).

In many classical experiments for the determination of JNDs, synthetic stimuli like vibrotactile displays are utilized to vary the sensory input across a wide perceptual range, which allows for a reliable estimation of individual perception thresholds. Our study focuses on real-world materials and friction perception in active exploration, which is influenced by factors such as skin moisture, movement speed, and individual physiology, as we found a significant correlation between the deformability *R*0 and the average relative difference in friction (r=0.43,p=0.014), but not for the elasticity *R*2 or the fast deformability *Ue* (r=-0.16,r=-0.15,p>0.37, respectively). In general, participants with lower median friction contrast used higher median normal force (r=-0.62,p<0.001). These influences create large variability between participants, making the task easier for some and harder for others, as shown in Fig. 8f. Participants with lower median $\Delta \mu /\mu$ tend to achieve a lower success rate in the friction ranking task (r=0.52,p<0.001). As a result, individual JNDs are difficult to determine with one sample set, and our analysis focuses on group-averaged thresholds using the combined answers of all participants.

We observed that ID20’s sorting of the microstructured samples showed the strongest positive correlation with the measured friction (r = 0.75, p = 0.029). However, this observation does not remain significant after correcting for multiple comparisons. Note that the roughness sorting of ID20 was negatively correlated to most other participants. Participants were repeatedly told that the sorting in Experiment I should be in respect to roughness, and the participants repeated this instruction. This suggests that, for some participants, distinguishing roughness from friction may be difficult.

### Discussion of Experiment III

Fingertip friction is known to depend on multiple factors, including the exploration movements (Zhou, Mo, Li, et al., 2020), skin moisture (Adams, Johnson, Lefévre, et al., 2013; Fehlberg et al., 2024; Infante et al., 2025, the duration of exploration (Adams et al., 2013), and material properties like the surface texture (Saikumar et al., 2025; Skedung et al., 2011). Nevertheless, participants are reliably able to judge friction differences between textured surfaces. We found a JND of approximately 20 % for the friction coefficient on the MS samples which is consistent with our previous findings for similar surface textures (Fehlberg et al., 2022).

Transient changes in friction have been detected even for smaller differences (11 %) (Gueorguiev et al., 2017) than those observed here. The larger JND may arise from confusion brought about by the surface structure and the more difficult task, which requires the participant to lift the finger and change between textures, whereas the transient changes could be detected with the finger remaining in sliding contact.

The texture size difference between samples in a comparison pair has an interesting influence on friction discrimination performance. Within sample groups of similar texture sizes (intra-group A and intra-group B), participants detected smaller friction differences (17 %) compared to friction differences between samples across the two groups (inter-group A and B, 23 %). This pattern supports the idea that texture size differences can interfere with friction discrimination. In line with the duplex theory of tactile texture perception, the grouping of samples would then correspond to a division into fine- and coarse-textured surfaces. We suggest that the principles of the duplex theory may also play a relevant role in friction perception, which was previously suggested in Refs. (Fehlberg et al., 2022; Skedung et al., 2020). In this picture, friction perception of fine-textured surfaces would build on temporal and spatial cues, while friction perception of coarse-textured surfaces would be judged by spatial cues.

Texture-dependent vibrations during sliding may additionally contribute to friction perception (Fagiani, Massi, Chatelet, et al., 2011). Future work could combine psychophysical friction measurements with direct recordings of vibration signals to better understand their contribution to the observed differences in friction discrimination.

The role of normal force in friction perception is not yet fully understood. While friction force increases with normal force, it has been suggested that this relationship is not purely linear as expressed in Amontons’ law. The functional relationship between friction and normal force may depend on properties of the explored material and texture as well as on the normal force regime (Inoue et al., 2022; Tomlinson, Lewis, & Carré, 2009). Previous research has shown that friction perception does not rely only on the absolute friction force. Instead, the applied normal force is integrated into the friction judgments, demonstrating a perceptual constancy in friction perception (Fehlberg et al., 2024). Since the JND values in the present study are consistent with those in Ref. (Fehlberg et al., 2022), where the use of normal force was constrained, we further suggest that restricting normal force within ranges typically used by participants in free exploration tasks does not change the ability to judge the strength of friction.

To summarize Experiment III, we find that participants use different tactile exploration parameters such as speed and normal force, when the task left them to choose. While pillar diameter and volume showed a strong correlation with perceived roughness, they did not show a correlation with measured friction. The friction differences between samples were perceived when greater than 20 %, which confirms findings in Ref. (Fehlberg et al., 2022). The most important conclusion pertains to an extension of the duplex theory of touch to friction perception. The performance in friction discrimination was better between samples with either only coarse or only fine texture than between samples with one coarse and one fine textured surface. We suggest that the dependence of friction perception on spatial and temporal cues for textures larger and smaller than 100 µm is similar to that of roughness perception. Similar to previous results for tactile perception of various materials, friction perception of microstructured rubber samples is entangled with roughness perception.

## General discussion

In the present study we investigated the perception of roughness, texture and friction in three separate experiments. We discuss how these perceived properties relate to one another and in how far they influence each other.

### Roughness and texture perception

A central finding of this study is the strong correlation between geometrical surface parameters and participants’ roughness judgments. Namely, measured roughness of randomly rough surfaces and pillar dimensions of periodically microstructured surfaces predict participants’ ratings of roughness and texture. Under dynamic tactile exploration, participants did not systematically differentiate between periodic and random surface textures, indicating that texture and roughness led to a similar percept across both sample sets. This result aligns well with the framework proposed by Okamoto, Nagano, and Yamada (2013), in which tactile roughness perception is structured along fine and macro scales, without explicit differentiation between periodic and random textures.

The lack of perceptual differentiation between periodic and random textures contrasts with the absence of physical parameters that can be consistently applied to both surface types, as commonly used metrics are often specific to either randomly rough or periodic structures. With Experiment Ic, we address this gap by employing standardized roughness measures ($R_q$, $R_z$, and $R_{\Delta q}$) that allow a joint physical description of perceptual roughness across structurally distinct surfaces.

Participants agreed slightly less in the texture rating experiment than in the roughness sorting task. We attribute this difference to the difficulty of the tasks. Rating texture on a continuous scale is likely more abstract and requires the participants to memorize samples they touched before, which can be associated with higher cognitive demands than the sorting task.

In all three experiments, we found indications of perceptual differences between the four smallest and the four largest microstructures. Two different sample clusters of these samples were identified when analyzing the sorting results of Experiment I. In Experiment II, the samples in each of these clusters caused different texture perception when comparing static and dynamic tactile exploration. Finally, in Experiment III, we found significant differences in friction discrimination between sample pairs from one cluster and sample pairs drawn from different clusters. This indicates that the structural differences underlying roughness perception likewise affect friction perception, suggesting that both dimensions are not independent but perceptually entangled.

### Entanglement between roughness and friction perception

As entanglement between roughness and friction perception has been discussed in the literature (Janko et al., 2018; Okamoto et al., 2013; Skedung et al., 2020), we further quantified their relationship. Our dataset provides a unique opportunity to link perceived roughness and measured friction to friction perception, as both perceptual judgments were collected on the same set of samples and with comparable participant groups.

For the perceived roughness, we rely on the PSE values obtained in Experiment Ic, in an application of the method used to derive a roughness metric from the participants’ psychophysical judgments. To compare the perception of friction with its physical measurement, we estimated a perceptual friction scale using the Bradley-Terry model. For this analysis, we excluded comparisons with samples 60/120 and 150/350 to obtain the same occurrence of all samples in the pair comparisons. Based on these pairwise comparisons, we constructed a combined decision matrix for all participants, reflecting how often the friction of each sample was chosen over that of another. Using an iterative estimation procedure, we derived a perceptual scale representing the relative friction perception across all samples. This perceptual scale is plotted against the measured friction in the SI in Fig. S9. This perceived friction scale is significantly correlated with the average normalized friction coefficient (r=0.936,p=0.006), indicating that the participants indeed perceived friction differences reliably.

A linear regression model for perceived friction was computed using measured friction and perceived surface roughness (perceived $R_{\Delta q}$ from Experiment I) as predictors. The model significantly explained variance in perceived friction ($R^2~=~0.973, R^2_{\text {adj.}}~=~0.955, F~=~54.27, p~=~0.004$). Measured friction showed the strongest positive effect (β=13.27,p=0.002), while $R_{\Delta q, \text {perc.}}$ contributed with a smaller but still significant effect (β=3.94,p=0.046). While measured friction alone explains 87.6 % of the perceived friction ($R^2=0.876$, $R^2_{\text {adj.}}=0.845$), the perceived roughness accounts for an additional 9.7 % of the explained variance in friction perception.

Our results indicate that, on average, approximately 10% of the variance in friction perception can be explained by perceived roughness. While some participants seem capable of distinguishing the two perceptual dimensions more clearly, others (for example, ID20) exhibit a stronger mutual influence of friction and roughness in their judgments. At the same time, the presence of two distinct clusters in friction judgments suggests extending the duplex theory of tactile texture perception to friction perception.

### Duplex theory of texture and friction perception

The duplex theory proposes that the tactile perception of texture relies on two distinct perceptual pathways, one based on spatial cues and one based on temporal cues, which are mediated by different types of mechanoreceptors in the glabrous skin. We found two sample clusters in all three perception experiments, and propose that their separation reflects the prevalence of one of the two perceptual channels, each linked to a dominating mechanoreceptor type. This is consistent with the idea that the separation into spatial and temporal perceptual regimes is not limited to rigid textures, but generalizes to compliant surface structures.

During the dynamic exploration of materials, characteristic vibrations at frequencies *f* are induced into the skin, that depend on the spatial periodicity of the texture and the sliding velocity. Using the relation $f= v/p_s$ from Ref. (Bensmaia and Hollins, 2003) with *v* as the sliding speed and $p_s$ as the spatial periodicity, we can calculate the expected vibration frequencies induced by the dynamic exploration of our samples. With a median sliding speed of 50 mm/s and a spatial periodicity of twice the pillar diameter, the frequencies are 625, 416.7, 250, and 166.7 Hz for the diameters 40, 60, 100, and 150 µm, respectively. The vibrations do not depend on the pillar height, in agreement with our observation that pillar diameter explains most of the roughness perception without pillar height adding information.

Tactile perception is the result of processing input of all types of mechanoreceptors, and there is complementary evidence on which mechanoreceptor may contribute predominantly for which textures. With an ability to respond up to 1000 Hz (Quindlen, Stolarski, Johnson, et al., 2016), the expected dominant frequencies of our samples fall within the sensitivity range of Pacinian corpuscles (FA-II). Blake, Hsiao, and Johnson (1997) conducted a neurophysiological study in monkeys using plastic surfaces composed of raised dots with diameters between 250 and 2500 µm and heights between 280 and 620 µm. They showed that fast adapting receptors (FA-I) respond to all textures, but that only the responses of slowly adapting (SA-I) monkey afferents accounted for the psychophysical judgments by humans for the same coarse textures. In line with these findings, we expect that all our samples activate FA-I and FA-II receptors by sliding-induced vibrations. However, based on the results by Blake et al. (1997), we suggest that spatial and vibrational encoding of all afferents contribute to the perception of friction on the textures. The samples in group A fall into the texture sizes that Bensmaia and Hollins (2003) investigated. They found the vibration intensity to be the main cue in perception for fine textures and linked this cue to the activation of Pacinian corpuscles (FA-II).

Since the sample clusters identified in roughness and texture perception were relevant to results on friction perception, we now discuss the suggestion that friction perception relies not only on texture but also on two different perceptual pathways. Skedung, Harris, Collier, et al. (2020) concluded that friction is closely linked to the spatial aspect of touch perception because their analysis grouped friction in one tactile dimension with surface shape parameters such as peak height and slope. In this view, friction contributes to the quasi-static deformation-based channel of perception, which is usually attributed to the perception of coarse textures. The second tactile dimension of their study reflected vibration-related information generated during sliding, but did not align with friction.

These vibrations originate directly from frictional interaction and are defined by surface texture, sliding speed, and contact force (Fagiani, Massi, Chatelet, et al., 2012). Friction-related vibrations can be recorded as fluctuations in the tangential force during exploration, and have been related to tactile perception of pleasantness (Klöcker et al., 2013). It is thus reasonable to assume that vibrations play a role in the perceptual evaluation of friction. It was already suggested by Bensmaia and Hollins (2005) that not only roughness but also friction perception of fine textures rely on vibrational cues.

In the tribology of fingertip-material contacts, we find additional mechanical reasoning for a separation in spatial deformation and vibrational friction fluctuations. According to the review by Derler and Gerhardt (2012), human skin friction consists of two fundamental components: adhesive friction that arises from adhesion at microscopic contact points and dominates on fine roughness, whereas deformation friction results from viscoelastic skin deformation on coarse textures. The contribution of these two mechanisms depends on texture scale, they therefore form two regimes of frictional response.

Taken together, these findings indicate that friction comprises a quasi-static deformation component that aligns with the spatial pathway of the duplex theory, and an adhesive component that generates high-frequency micro-slip vibrations, aligning with the temporal pathway. This concurrence of psychophysical and tribological evidence suggests that the duplex theory, originally developed for texture perception, can also provide a meaningful framework for friction perception.

## Supplementary Information

Below is the link to the electronic supplementary material.

Supplementary file 1 (pdf 7431 KB)

## Data Availability

Not applicable

## Declarations

### Open Practices Statement

The raw datasets generated during the current experiments are available from the corresponding author on reasonable request. Data for Experiment I, II, and III are published: Fehlberg, M., & Bennewitz, R. (2026). Aggregated data set for the manuscript “Tactile Perception of Roughness, Texture, and Friction: The Duplex Theory Revisited” [Data set]. Zenodo. https://doi.org/10.5281/zenodo.20271426. None of the experiments were preregistered.
