# Supplementary material for: Tactile perception of roughness, texture, and friction on compliant surfaces
Source: Atten Percept Psychophys. 2026 Nov 11;88(8):224. doi: 10.3758/s13414-026-03361-1 (PMC13755061; doi:10.3758/s13414-026-03361-1)
Supplement: Supplementary file 1 [file 13414_2026_3361_MOESM1_ESM.pdf]

Supplementary Information for "Tactile Perception of Roughness, Texture, and Friction on Compliant Surfaces"

Maja Fehlberg[1,2] and Roland Bennewitz[1,2*]

[1]INM - Leibniz Institute for New Materials, Campus D2 2, Saarbrücken, 66123, Germany.
[2]Department of Physics, Saarland University, Campus, Saarbrücken, 66123, Germany.

*Corresponding author(s). E-mail(s): roland.bennewitz@leibniz-inm.de;

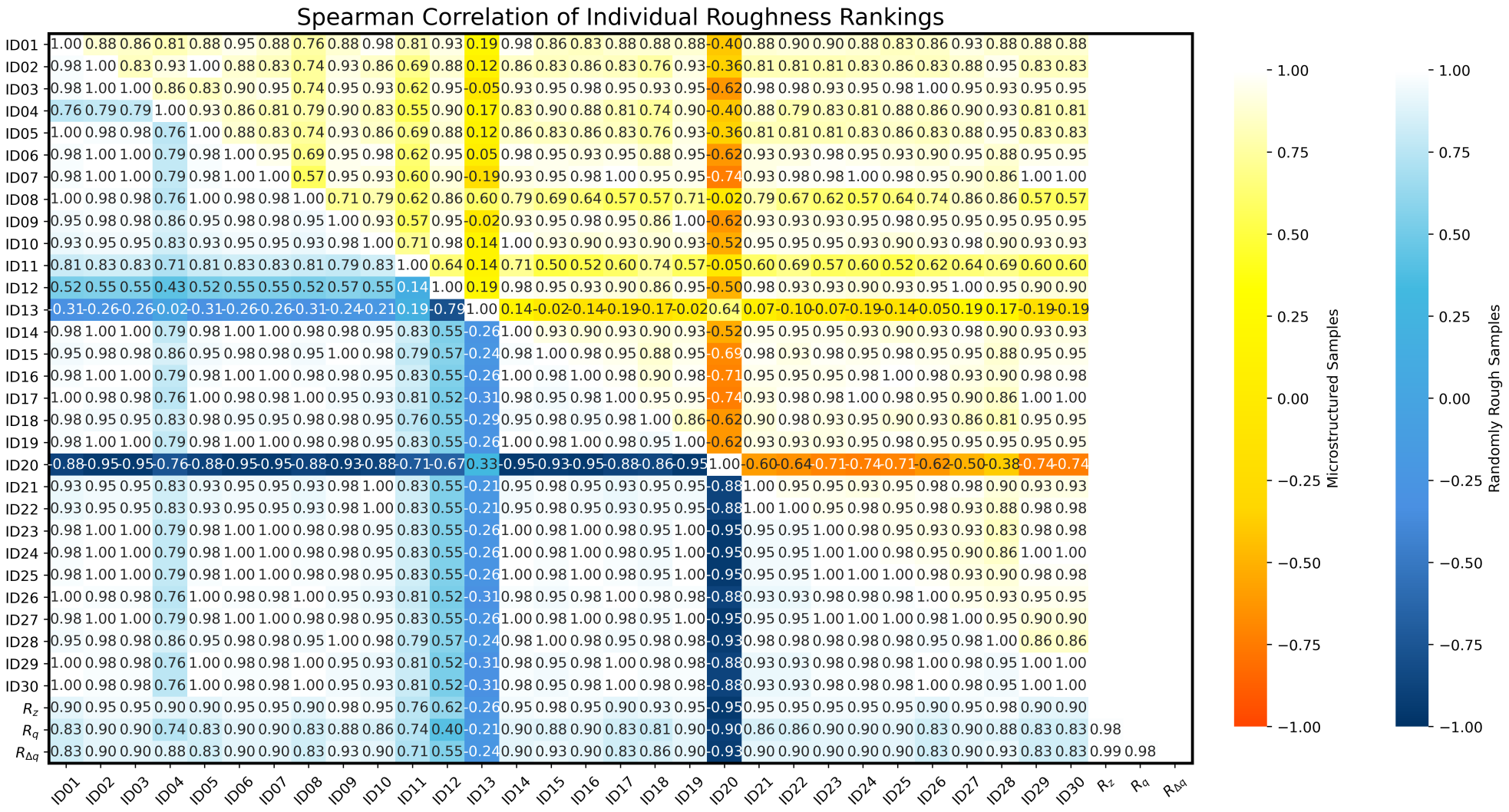

S1: Spearman Correlation Matrix for normalized perceived roughness scale for the randomly rough samples (lower left, blue color map) and the microstructured samples (upper right, orange color map) resulting from the *sorting* task. The last three rows show the Spearman correlation of the normalized PRS with the measured roughness values R_z, R_q and $R_{\Delta q}$.

1 Influence of Sample Dimensions

As described in the main text in section Experiment Ia & Ib, the participants were asked to place the eight samples in a line of 550 mm. Because of the sample dimensions (50 mm x 50 mm) only 150 mm space in total could be distributed while sorting. To ensure that the sample dimensions did not influence the perceived roughness scales, we repeated the analysis for both sample sets as follows. To eliminate the influence of sample dimensions on the analysis, we calculated a pairwise distance matrix between samples (with a minimum distance of 1 mm), correcting for expected linear spacing. Based on these distance matrices, we then estimated one-dimensional sample positions for each participant using metric multidimensional scaling (MDS), constrained to a single dimension. Since the Spearman correlation for the dimension corrected perceived roughness scale ($\mathrm{PRS}_{\mathrm{corr.}}$) and the PRS is 1, the Spearman correlations between the participants remain how stated in the main text.

The Fisher-Z-transformed average over all 28 participants correlations changes for the RR samples marginally to 0.92, 0.95 and 0.94 for R_q, R_z, and $R_{\Delta q}$ respectively. For the MS samples the correlations change to 0.88, 0.88 and 0.96 for pillar-volume, bending stiffness and diameter. The individual PRS and $\mathrm{PRS}_{\mathrm{corr.}}$ are highly correlated with Fisher-Z-transformed average of $r = 0.989$ ($p < 0.001$) for MS samples and $r = 0.991$ ($p < 0.001$) for RR samples, which shows that the dimensions of the sample did on average not constrain the participants in their sorting task. Figure 2 and 3 in the main document were repeated with the dimension-corrected scales and provided in Figure S2 and S3. The clustering was repeated and we found an increased silhouette score for k = 2 with 0.59 and 0.57 for the microstructured and the RR samples.

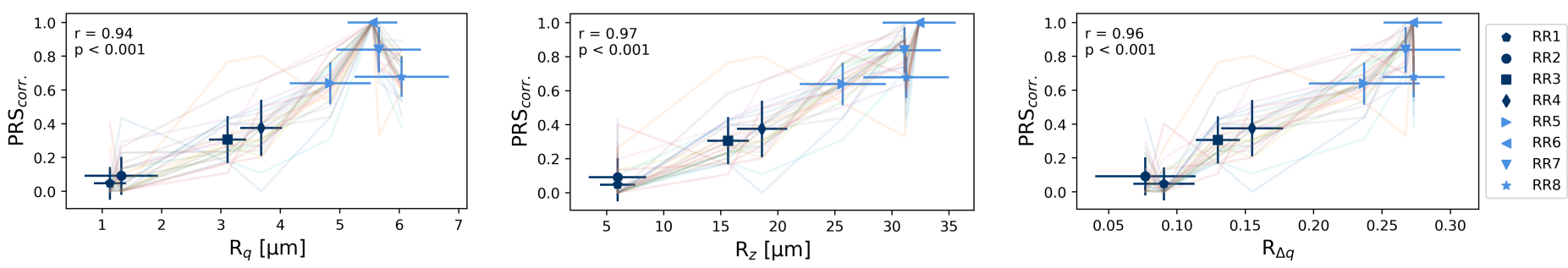

(a) (b) (c)

S2: Dimension corrected perceived roughness scales ($\mathrm{PRS}_{\mathrm{corr.}}$) as function of the measured roughness parameters R_q, R_z and $R_{\Delta q}$. Dots and error bars indicate the average $\mathrm{PRS}_{\mathrm{corr.}}$ with standard deviations. The Pearson correlations are reported within the figures. The dark and light blue dot colors indicate two clusters of samples identified by applying the k-means algorithm to the distance matrices of the participants' perception scales. Light colored lines report the individual PRS.

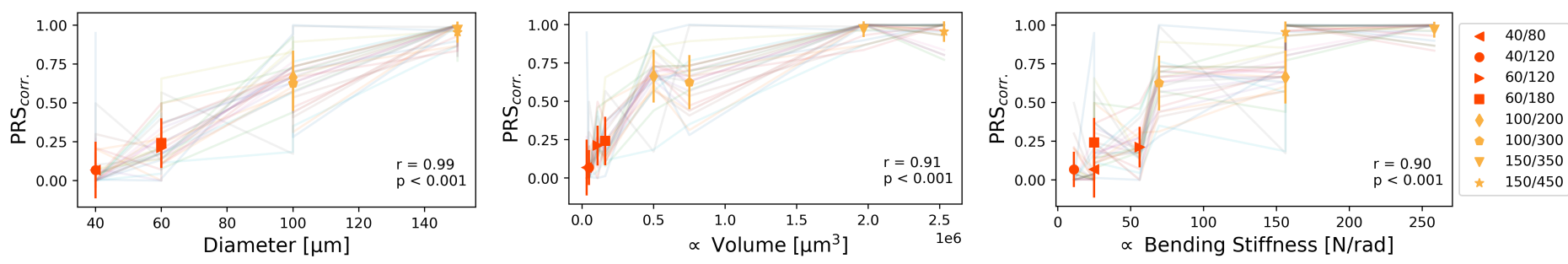

(a) (b) (c)

S3: The dimension corrected average perceived roughness scale for the microstructured samples is plotted (a) against the pillar diameter, (b) proportional to the pillar volume and (c) proportional to the bending stiffness. The light lines indicate the individual perceived roughness scales with colors representing different participants. The dark and light orange data points represent two sample-clusters that were found with k-means clustering.

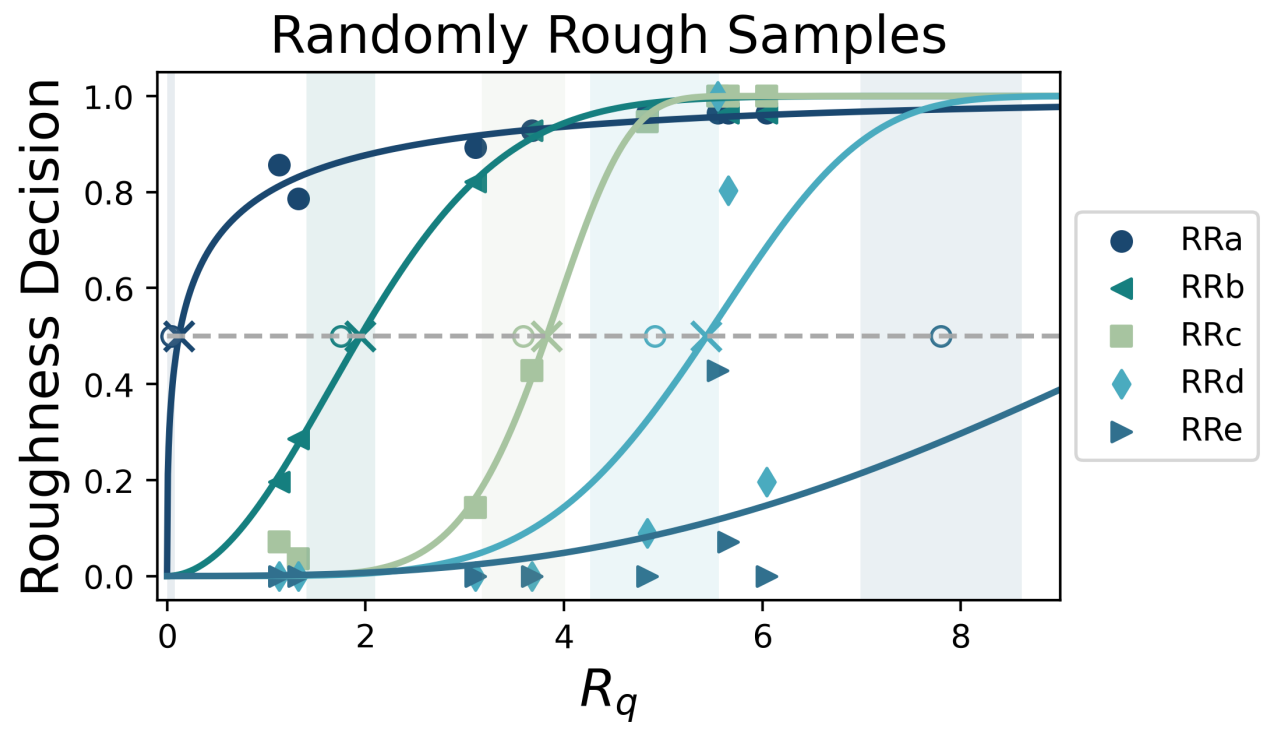

(a)

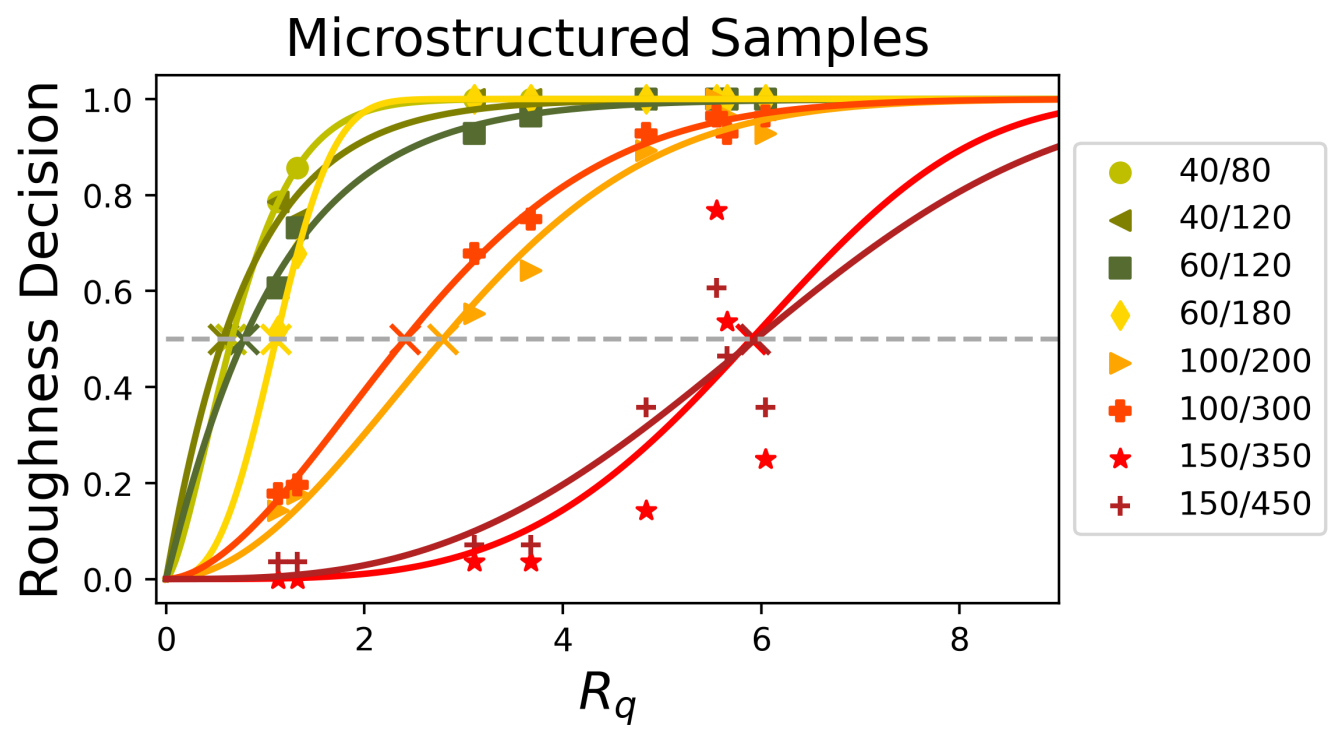

(b)

S4: Average roughness decisions derived from the inserting task in Exp. Ic. (a) Results for the five control RR samples. Hollow dots represent the measured R_q for the five control samples, the colored sections represent the standard deviation for this measurement. (b) Results for the microstructured samples. In both figures the crosses mark the point of subjective equality.

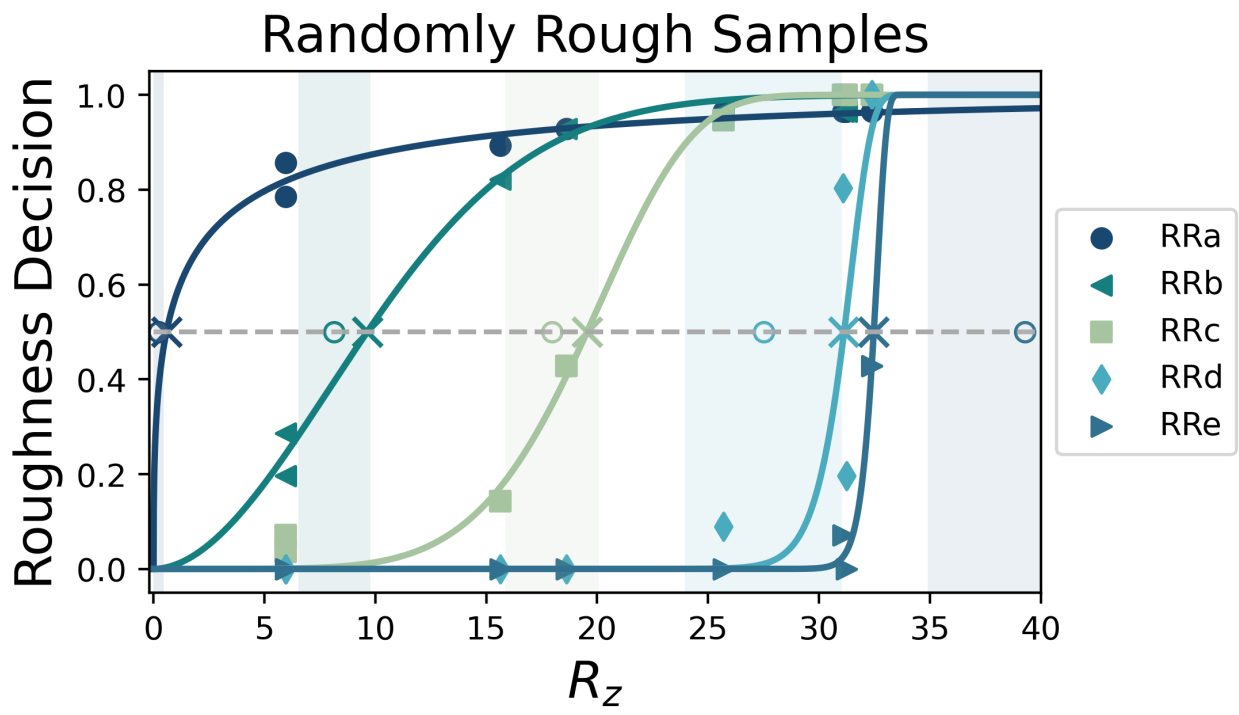

(a)

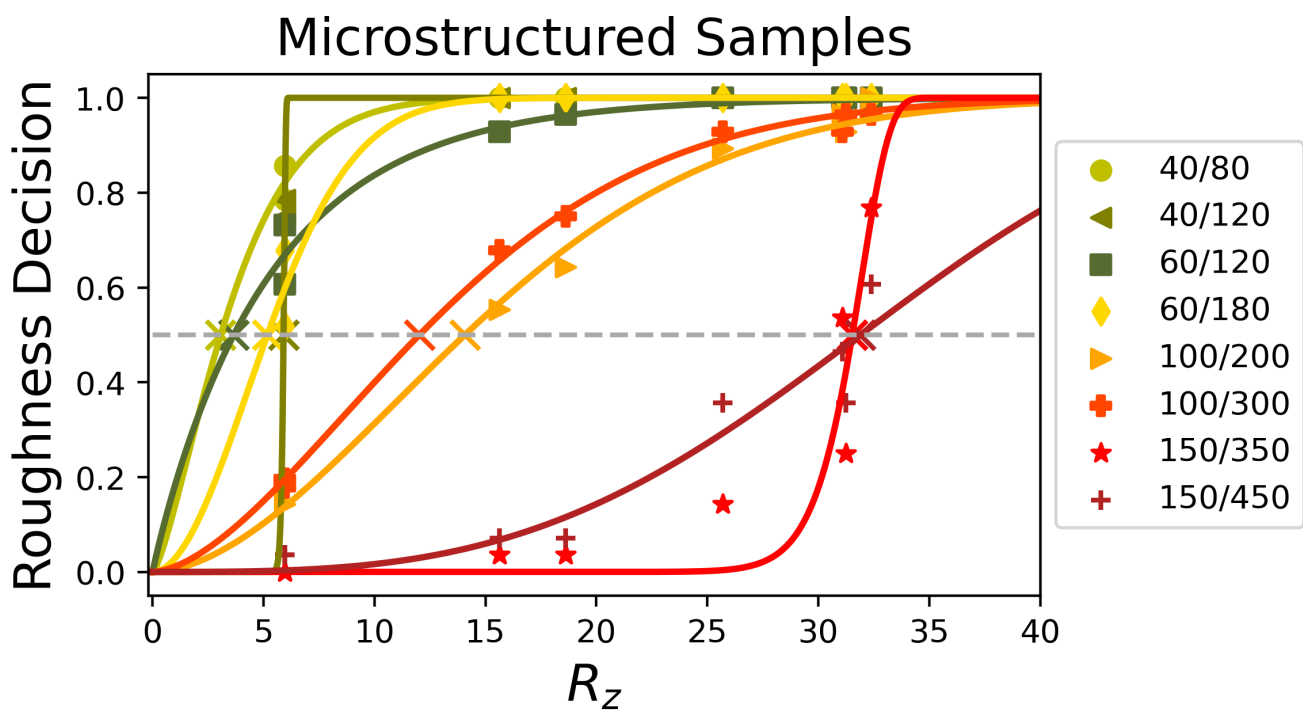

(b)

S5: Average roughness decisions derived from the inserting task in Exp. Ic. (a) Results for the five control RR samples. Hollow dots represent the measured R_z for the five control samples, the colored sections represent the standard deviation for this measurement. (b) Results for the microstructured samples. In both figures the crosses mark the point of subjective equality.

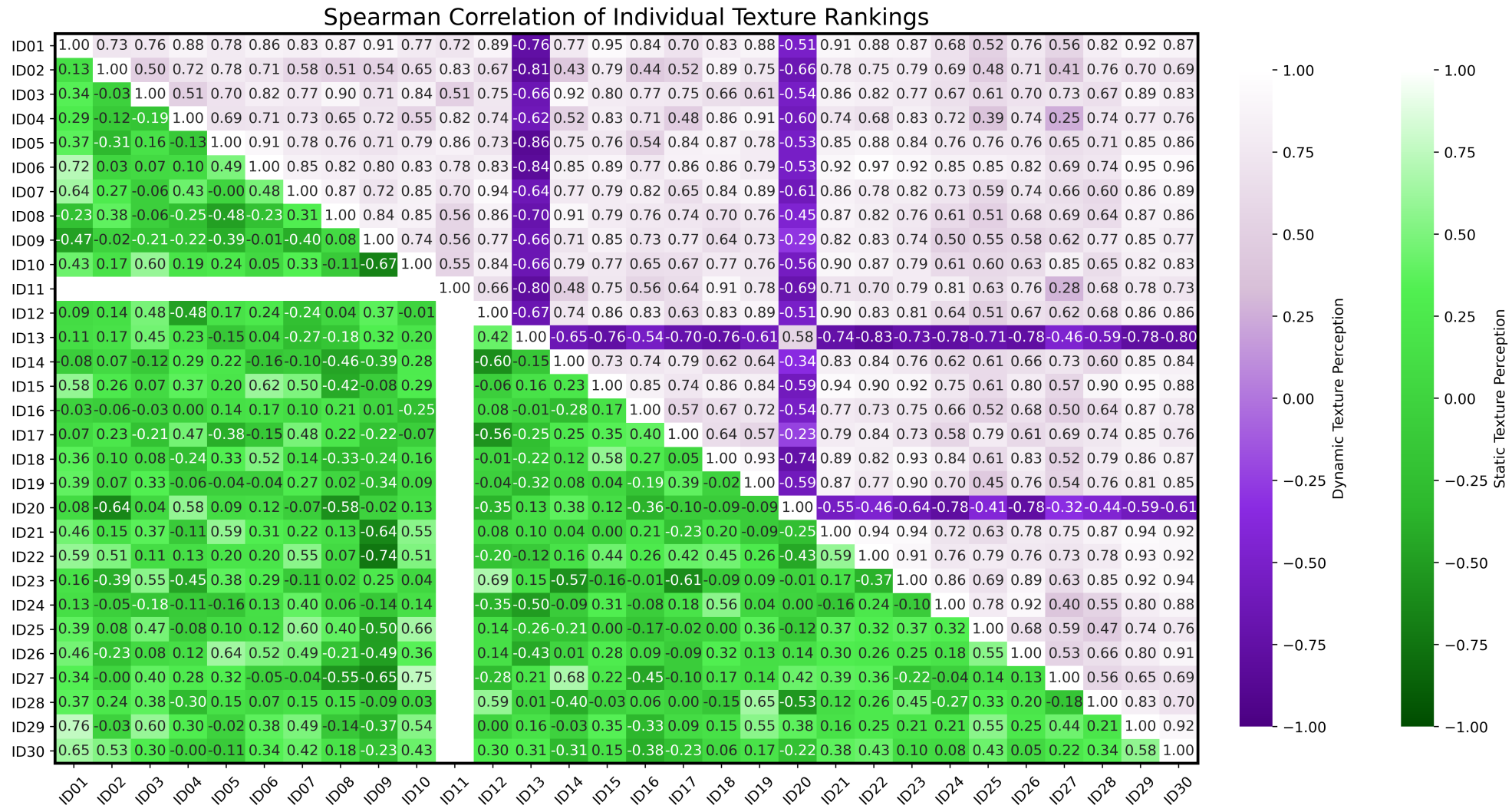

S6: Spearman Correlation Matrix for texture ratings for static (lower left, green color map) and dynamic (upper right, purple color map) exploration resulting from Experiment II.

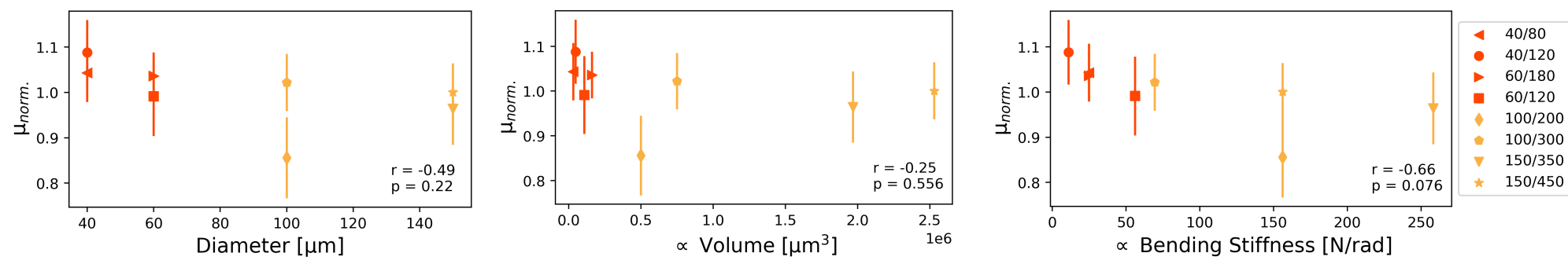

(a) (b) (c)

S7: The average normalized friction coefficient of the microstructured samples as function of (a) the pillar diameter, (b) the pillar volume and (c) the bending stiffness. Samples from group A are represented by dark and samples from group B by light orange markers.

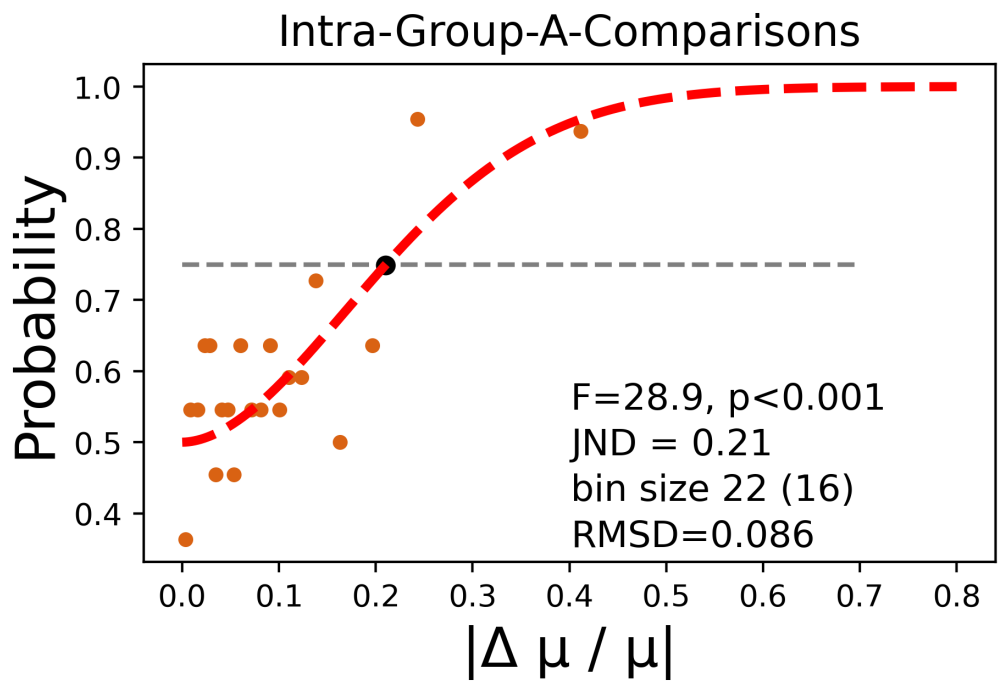

(a)

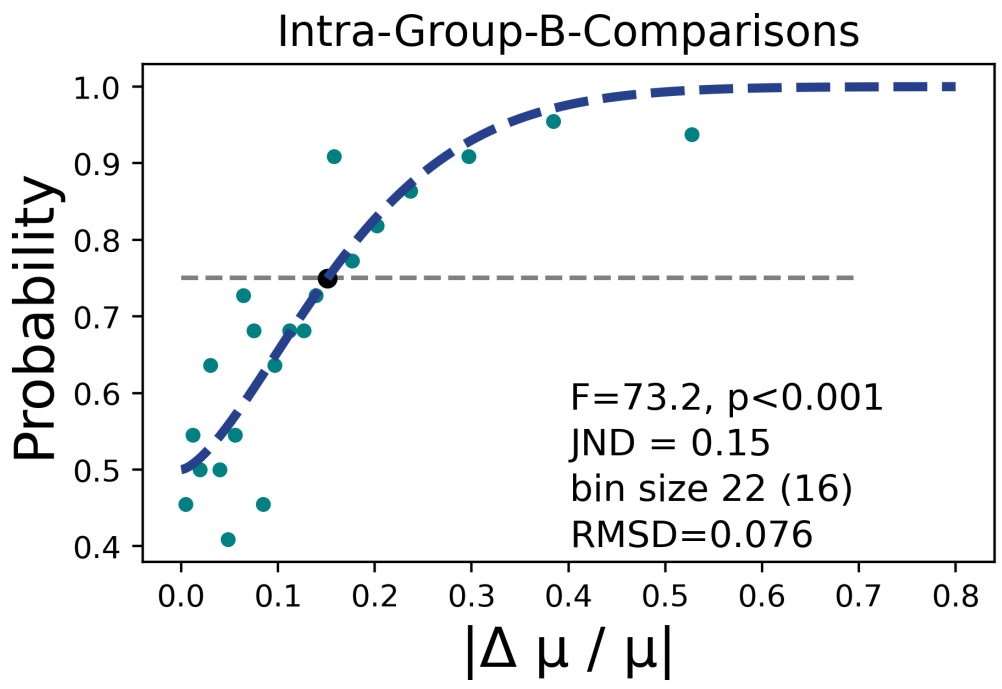

(b)

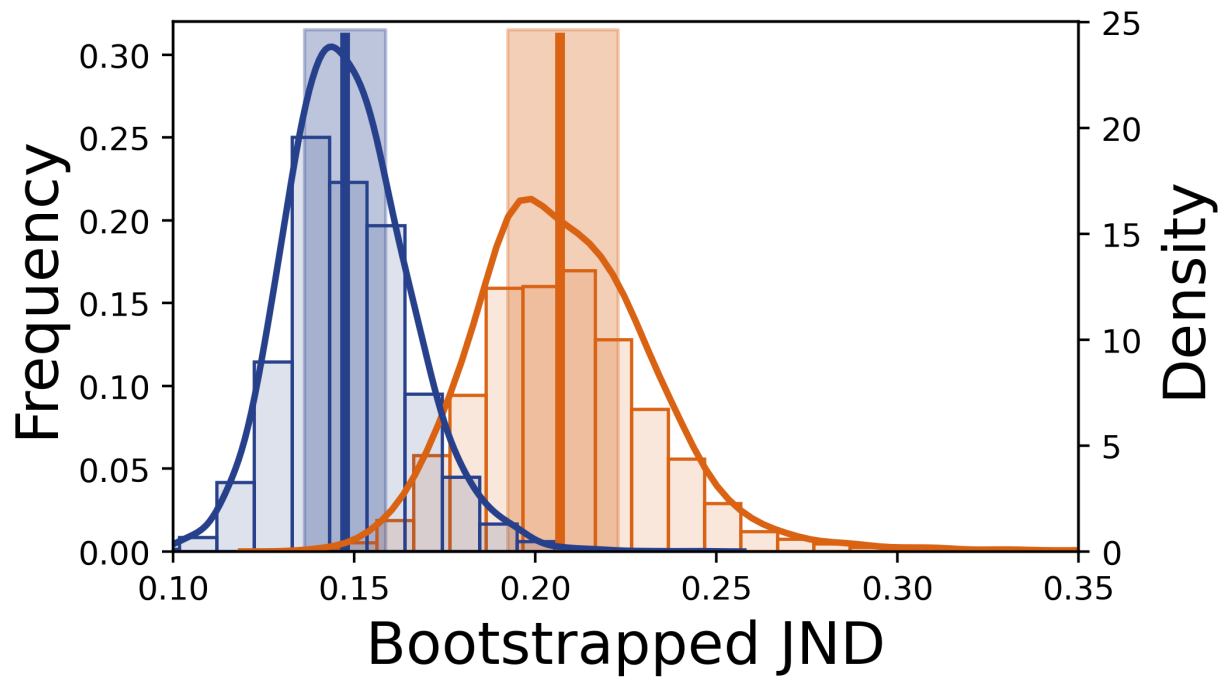

(c)

S8: a) and b) The probability that participants answered in agreement with the measured coefficient of friction is plotted against the relative difference in friction coefficient $|\Delta\mu/\mu|$. a) Summary of all sample comparisons within group A. b) Summary of all comparisons within group B. c) Histograms of JNDs calculated from bootstrapped datasets for both intra group A (orange) and intra group B (darkblue) comparisons. The medians, IQS and KDE are marked with the same color scheme.

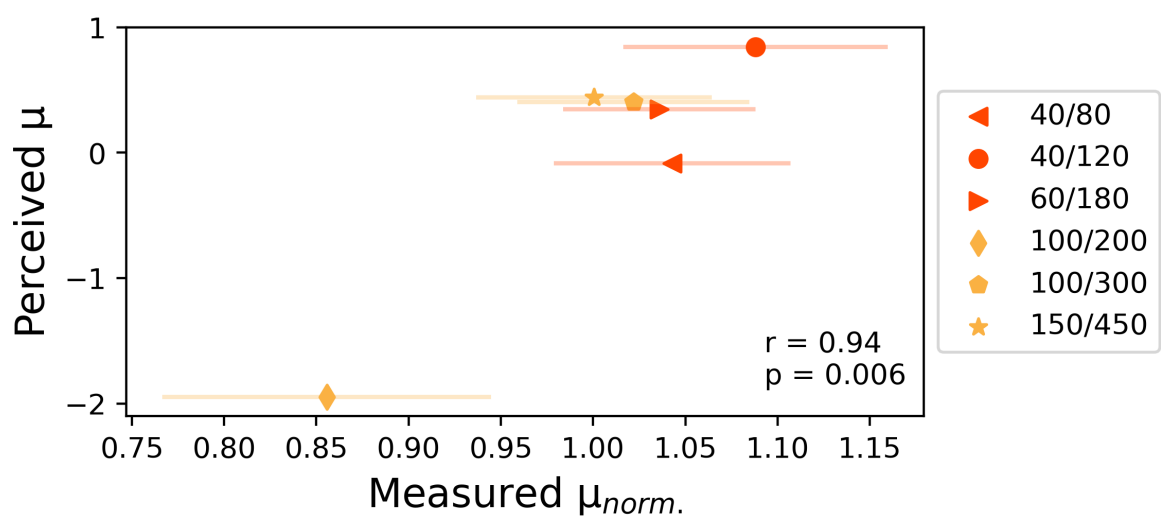

S9: The perceived friction estimated from Experiment II is plotted against measured friction.
